# HD_BPMDS: a curated binary pattern multitarget dataset of Huntington’s disease–targeting agents

**DOI:** 10.1186/s13321-023-00775-z

**Published:** 2023-11-17

**Authors:** Sven Marcel Stefan, Jens Pahnke, Vigneshwaran Namasivayam

**Affiliations:** 1 Drug Development and Chemical Biology, Lübeck Institute of Experimental Dermatology (LIED), University of Lübeck and University Medical Center Schleswig-Holstein, Ratzeburger Allee 160, 23538 Lübeck, Germany; 2 Department of Pathology, Section of Neuropathology, Translational Neurodegeneration Research and Neuropathology Lab, University of Oslo and Oslo University Hospital, Sognsvannsveien 20, 0372 Oslo, Norway; 3 School of Medical Sciences, Faculty of Medicine and Health, The University of Sydney, Camperdown, NSW 2006 Australia; 4 Department of Pharmacology, Faculty of Medicine, University of Latvia, Jelgavas Iela 4, Rīga, 1004 Latvia; 5 Department of Neurobiology, The Georg S. Wise Faculty of Life Sciences, Tel Aviv University, 6997801 Tel Aviv, Israel; 6 Department of Pharmaceutical and Cellbiological Chemistry, Pharmaceutical Institute, University of Bonn, An Der Immenburg 4, 53121 Bonn, Germany

## Abstract

The discovery of both distinctive lead molecules and novel drug targets is a great challenge in drug discovery, which particularly accounts for orphan diseases. Huntington’s disease (HD) is an orphan, neurodegenerative disease of which the pathology is well-described. However, its pathophysiological background and molecular mechanisms are poorly understood. To date, only 2 drugs have been approved on the US and European markets, both of which address symptomatic aspects of this disease only. Although several hundreds of agents were described with efficacy against the HD phenotype in in vitro and/or in vivo models, a successful translation into clinical use is rarely achieved. Two major impediments are, first, the lack of awareness and understanding of the interactome—the sum of key proteins, cascades, and mediators—that contributes to HD initiation and progression; and second, the translation of the little gained knowledge into useful model systems. To counteract this lack of data awareness, we manually compiled and curated the entire modulator landscape of successfully evaluated pre-clinical small-molecule HD-targeting agents which are annotated with substructural molecular patterns, physicochemical properties, as well as drug targets, and which were linked to benchmark databases such as PubChem, ChEMBL, or UniProt. Particularly, the annotation with substructural molecular patterns expressed as binary code allowed for the generation of target-specific and -unspecific fingerprints which could be used to determine the (poly)pharmacological profile of molecular-structurally distinct molecules.

**Supplementary Information:**

The online version contains supplementary material available at 10.1186/s13321-023-00775-z.

## Objective

### Drug annotation

The acquisition, comprehension, and utilization of integral chemical associations are essential for modern drug development. Drug annotation and profiling are important measures to gain a wealth of data to predict structurally and functionally distinctive novel drug candidates—particularly in the light of modern machine learning (ML), neural networks (NNs), and artificial intelligence (AI) approaches. Curated high-quality datasets of annotated drugs and other small-molecule ligands allow for the determination of molecular-structural and physicochemical requirements to trigger a desired biological response, and thus, advanced computational model development in modern drug discovery.

### Data bias

Proper drug annotation depends on several crucial factors that determine the goodness of the data used. The accessibility and readability of chemical structures, for example, is a huge obstacle in chem- and bioinformatics until today. Most chemistry-related articles are available in print only, and optical recognition tools for both text and chemical structures are necessary to transform the data for machine use [1, 2]. These tools are still in their infancy today. Furthermore, only a very small number of journals require molecular-structural information, for example, SMILES codes [3]. Thus, public chemical databases that provide large-scale information on drug annotation rely on the goodwill of authors, librarians, and the performance of the optical chemical recognition tools.

Another very important factor is the biological background of the assays used to determine bioactivity. Most data were generated with high-throughput screening (HTS) assays using single point measurements only. These data are often not supported by alternative assays and full-blown concentration-effect curves. HTS is prone to assay artifacts due to compound-, assay-, or cell line-/host system-related unspecific effects. Complementary experiments to support or disprove initial findings are, unfortunately, required for a minority of journals only [4]. This leads inevitably to pollution of the chemical landscape with incorrect annotations. This lack of complementation reaches further than the individual protein of interest. As resources are limited in virtually every laboratory, assays beyond the target of interest are barely performed, which accounts even more for other target classes that may be of relevance. This leads to the annotation of many molecules as ‘selective’ or ‘specific’ although the truth about these attributes is unknown.

Finally, chemical databases such as PubChem (https://pubchem.ncbi.nlm.nih.gov) pose themselves a risk of misinterpretation. Millions of annotated molecules are available. However, these are in most cases not validated, and once stored publicly, the information (also incorrect annotations or the lack of annotations) are saved forever. Validated datasets, such as ChEMBL (www.ebi.ac.uk/chembl/) exist, however, the validation process strongly reduced the small-molecule landscape, and thus, the molecular-structural diversity and opportunity space are also limited. In addition, although drug-gene, drug-target, or drug-disease annotations are implemented in most public databases, the true polypharmacology of these molecules remains hidden as molecules are stored on a ‘one target-one molecule’ basis only. In summary, these data biases hamper modern drug development approaches [5].

### Multitarget datasets

The above-named aspects make a manual compilation and curation of data necessary to properly study the molecular coherences of particular diseases and to create novel networks of interoperable data. This is even more evident for orphan diseases, such as Huntington’s disease (HD), for which a general sparseness of data is given. Multitarget datasets (MDSs) support medical research to identify target classes and/or constellations underlying a certain pathological condition. MDSs correlate bioactivity landscapes of small-molecules toward different related or unrelated pharmacological targets which is crucial for novel drug design and discovery.

Recently, we reported on an MDS focusing on the ATP-binding cassette (ABC) transporters ABCB1, ABCC1, and ABCG2 that correlated substructural molecular components of small-molecule inhibitors to their effect on these three targets [6]. These substructures were presented as binary code, making their processing easy-to-use. Applying a self-developed computational prediction tool—‘computer-aided pattern analysis’ (‘C@PA’)—we were able to predict structurally distinctive and potent inhibitors of these three targets by a prediction rate of 21.7% [7]. Validation of C@PA by overcoming structural [8] and bioactivity [9] limitations resulted in an even higher hit rate of 40.0%. The high hit rates of C@PA allowed for fairly low numbers of in vitro-analyzed compounds [7–9], offering a positive perspective for research groups with financial constraints, and thus, global applicability. The inclusion of physicochemical parameters into this MDS allowed for their correlation to bioactivity, which was biologically confirmed [10]. Thus, molecular patterns are important tools in novel drug design and development.

### Rationale

Here, we report an MDS of 429 HD-targeting small-molecules that demonstrated efficacy in in vitro and/or in vivo HD models. These 429 small-molecules were analyzed for molecular substructures resulting in 261 active (= present) substructures in a statistical binary pattern distribution scheme. Molecular-structural and physicochemical descriptors as well as benchmark database-linked identifiers complement the HD binary pattern MDS (HD_BPMDS). The HD_BPMDS poses five major advantages:

(i) One major impediment of previous HD therapy attempts was the strict adherence to the ‘specificity paradigm’—the ‘one drug-one target principle’ [11] that did not lead to clinical success. Enabling the controlled engagement of several HD-related drug targets by polypharmacological agents poses a real chance to successfully address HD in the future. Pattern-based multitarget fingerprints derived from MDSs support the identification of novel molecular-structural entities to discover such agents;

(ii) The knowledge of substructural features that promote or impede polypharmacology allows for the design and development of selective, single-targeting agents for analysis and diagnosis purposes, e.g., novel fluorescence- or positron-based imaging techniques to study the expression and/or function of key proteins;

(iii) Provision of the entire (successfully evaluated) target landscape enables the identification of repetitive targets that have frequently been addressed in independent studies. The association of these repetitive targets with drugs/small-molecule ligands promotes not only the identification of novel polypharmacological agents as outlined in (i), but also ensures the actual addressability of these apparent HD key players;

(iv) The entire landscape of (successfully evaluated) targets allows for the target-based expansion to yet uncovered, not with HD associated target proteins. Many of the stated target proteins and pathways are embedded in larger cascades that are known and/or a certain basic knowledge has been deduced from. Hence, under consideration of these cascades, cross-talks, and constellations, not only target space, but subsequently also the drug and small-molecule ligand space can be expanded.

(v) Apart from potential therapeutic (i) and diagnostic (ii) options and the establishment (iii) [and extension (iv)] of current (and future) therapeutic/small-molecule ligand-target protein(s) interaction(s), the elucidation of the modes of action of these therapeutics/small-molecule ligands and the underlying molecular mechanism of HD becomes more feasible under consideration of the given data.

The HD_BPMDS is freely available under the https://zenodo.org [12] URL, the https://panabc.info website [13], as well as under https://OSF.io [14], as its use is free of charge.

## Data description

### Data origin

Recently, we reported on a pilot MDS of HD-targeting agents (HD_MDS). It contained 358 unique molecules extracted from 151 reports of 86 journals. These molecules showed either efficacy against in vitro and/or in vivo HD models or have been under clinical evaluation as therapeutics against or diagnostics for HD [15]. We updated the dataset under consideration of a recently published review article [16] and associated original reports. This update extended the dataset by further 71 molecules from 38 reports from 30 journals. In total, 189 reports from 104 journals between 1984 and 2022 were taken into account covering the entire spectrum between medicinal chemistry, chemical biology, clinical pharmacology, and other multidisciplinary life sciences.

The 429 literature-retrieved compounds were visualized using ChemDraw Pro version 20.1.1.125, and important substructural elements such as aromatic or aliphatic rings, side chains, or certain elements were identified. These identified substructural elements were derivatized by scaffold fragmentation and substructure hopping as reported earlier [8] and the output substructures were stored in a substructure catalog as previously described [6]. The molecular-structural diversity and quantity of the substructure catalog was increased taking alternative datasets of ABC transporter modulators [17–19] into account. Applying the query search function of InstantJChem version 21.13.0, the substructure catalog was subjected to the 429 compounds by an individual pattern analysis [9], discovering 261 unique active substructures that occurred at least once within the dataset.

### Data records

The HD_BPMDS consists of:

(i) individual identifiers for each compound, particularly
  - a unique HD_MDS identifier for each compound (‘HD_MDS_0XXX’)
  - the original name of the compound as given in the original report(s)
  - a common abbreviation of the original name of the compound
  - an important synonym of the compound
  - an alternative synonym of the compound
  - the PubChem Compound ID retrieved from https://pubchem.ncbi.nlm.nih.gov (400 of 429 compounds)
  - the ChEMBL Compound ID retrieved from https://ebi.ac.uk/chembl (336 of 429 compounds)
  - the DrugBank Accession Number as retrieved from https://go.drugbank.com (181 of 429 compounds)
  - the IUPHAR/Guide to Pharmacology Ligand ID as retrieved from https://guidetopharmacology.org (164 of 429 compounds)
  - the Chemical Abstracts Service (CAS) number as retrieved from https://commonchemistry.cas.org (268 of 429 compounds)
  - the systematic compound name according to the IUPAC nomenclature generated by ChemDraw Pro version 20.1.1.125

(ii) molecular-structural and physicochemical descriptors, particularly
  - the molecular structure of the compound conserved as SMILES code obtained either from
    1. the PubChem database (https://pubchem.ncbi.nlm.nih.gov) or
    2. manual drawing using ChemDraw Pro version 20.1.1.125 according to the 2D representation as given in the respective report(s) and/or supplementary information file(s);
  (b) the chemical formula as determined by ChemDraw Pro version 20.1.1.125
  (c) the physicochemical properties as calculated with MOE version 2019.01:
    1. calculated octanol–water partition coefficient (CLogP)
    2. calculated solubility (CLogS)
    3. molecular weight (MW)
    4. molar refractivity (MR)
    5. topological polar surface area (TPSA)
  (d) molecular-structural properties as calculated by MOE version 2019.01:
    1. number of hydrogen-(H)-bond donors
    2. number of H-bond acceptors
    3. number of rotatable bonds
    4. number of heavy atoms

(iii) a binary code (1 = active; 0 = inactive) for each of the 261 molecular substructures of the substructure catalog including
  - an individual substructure identifier (‘Substructurwe_0XXX’)
  - number of hits within the 429 compounds sorted from most abundant (left) to most rare (right)
  - number of heavy atoms
  - number of defined/irreplaceable hydrogens (‘[H]’ in SMILES codes)
  - chemical structure represented as SMILES code
  - the trivial name of the substructures

(iv) the compound category or categories in which the 429 compounds were allocated in, i.e.,
  - pharmaceutical drug/diagnostic
  - drug-like compound/chemical substance
  - nutrient/metabolite

(v) the addressed pharmacological target(s) and/or pathway(s), i.e.,
  - the name of addressed targets 1, 2, …, and 8 or addressed pathway 1, 2, and 3
  - the mode(s) of action against target 1, 2, …, and 8
  - the abbreviation of the name of targets 1, 2, …, and 8
  - the UniProt ID/PubChem Protein ID of targets 1, 2, …, and 8 as retrieved from https://uniprot.org / https://pubchem.ncbi.nlm.nih.gov
  - the PubChem gene name and gene ID as retrieved from https://pubchem.ncbi.nlm.nih.gov
  - the ChEMBL Target ID as retrieved from https://ebi.ac.uk/chembl
  - the IUPHAR/Guide to Pharmacology Target ID as retrieved from https://guidetopharmacology.org
  - the other modes of action 1, 2, and 3
  - the associated pathways 1, 2, and 3
  - the effect on pathways 1, 2, and 3

(vi) the target category or categories of the addressed pharmacological target(s) or pathway(s) in which the 429 compounds were allocated in, i.e.,
  - neurotransmitter systems
  - mitochondrial systems
  - *muHTT* RNA or DNA
  - muHTT protein
  - novel targets
  - target category unknown

(vii) the drug development stage of the compounds, i.e.,
  - in vitro
  - pre-clinical/in vivo or pre-clinical diagnostic
  - clinical trial or case study
  - off-label use
  - approved to treat HD

(viii) the HD symptoms that were addressed, anticipated to address, or observed in clinical trials, case studies, or pre-clinical evaluations

(ix) the cellular HD models 1, 2, …, and 4 used to assess the compounds, particularly
  - the name of the cell line(s)
  - the species of the cell line(s)
  - the Cellular Passport ID(s) of the cell line(s) as retrieved from https://cellmodelpassports.sanger.ac.uk
  - the Cellosaurus ID(s) as retrieved from https://cellosaurus.org
  - the American Type Culture Collection (ATCC) ID(s) as retrieved from https://atcc.org

(x) the animal HD models 1 and 2 used to assess the compounds, particularly
  - the name of the animal model(s)
  - the species of the animal model(s)

(xi) the digital object identifiers(s) [DOI(s)] for reports not listed on PubMed (https://pubmed.ncbi.nlm.nih.gov) or the PubMed identifier(s) [PMID(s)] retrieved from the National Center for Biotechnological Information (NCBI; https://ncbi.nlm.nih.gov) of the original report(s).

## Curation

### Literature data

The original HD_MDS was generated by compiling 358 compounds that showed efficacy against in vitro and/or in vivo HD models or have been under clinical evaluation as therapeutics against or diagnostics for HD. For this purpose, the NCBI web page (https://ncbi.nlm.nih.gov) was searched for the key words ‘small-molecule’ and ‘Huntington’s’ to obtain relevant reports. From these reports, a first selection of both high-class review (e.g., [15, 20–24]) and research (e.g., [25, 26]) articles has been obtained from which a large number of molecules could already been retrieved from. These articles represented the backbone of the HD_BPMDS, and were used for deep literature mining of the introduction and reference sections taking the original reports (in review articles) or cross-references (in research articles) into account. Cross-validation comparing either several review articles or aligning the information of one review article with the original reports it was citing enabled for the identification and verification of critical aspects such as **(**i) the small-molecule agents itself; (ii) its addressed target(s), target category or categories, and mode(s) of action; (iii) the in vitro or in vivo model(s) used for its assessment including the description of the addressed/anticipated/observed HD phenotype; and (iv) its drug development stage.

### Small-molecule agents

The retrieved molecules from the deep literature mining were either taken from the PubChem database (https://pubchem.ncbi.nlm.nih.gov; e.g., commonly known drugs) or manually drawn applying ChemDraw Pro version 20.1.1.125 according to the 2D representation as given in the respective report(s) and/or the supplementary information file(s). Isomeric SMILES were considered where applicable to allow for the greatest possible stereochemical diversity of the dataset. If retrieved from PubChem, the respective 2D representation of the molecules as generated in ChemDraw Pro version 20.1.1.125 were compared to the 2D representation of the respective report for cross-validation purposes.

The 429 molecules were imported into the MarvinSketch editor implemented in InstantJChem version 21.13.0. The molecular structure was considered as valid in the case that the loaded SMILES code appeared as the intended original molecular representation without any errors. In a final validation step, all SMILES were searched for on the PubChem database (https://pubchem.ncbi.nlm.nih.gov) and the resultant hit compared to the initial 2D representation of the respective report. Finally, molecular structures were compared to each other, and duplicates were erased and their associated additional data merged with the already existing entry in the updated HD_MDS.

## FAIR‑ification

### Annotation

A prime criterion of interoperability of the given data is its multidimensional annotation to public databases. In order to achieve this, we cross-linked the given data with various identifiers of commonly known public databases:

(i) Compound annotation with
  - PubChem (https://pubchem.ncbi.nlm.nih.gov)
  - ChEMBL (https://ebi.ac.uk/chembl)
  - DrugBank (https://go.drugbank.com)
  - IUPHAR/Guide to Pharmacology
    (https://guidetopharmacology.org)
  - CAS (https://commonchemistry.cas.org)

(ii) Target annotation with
  - UniProt (https://uniprot.org)
  - PubChem (https://pubchem.ncbi.nlm.nih.gov)
  - ChEMBL (https://ebi.ac.uk/chembl)
  - IUPHAR/Guide to Pharmacology
    (https://guidetopharmacology.org)

(iii) Cell model annotation
  - Cell Model Passports
    (https://cellmodelpassports.sanger.ac.uk)
(b) Cellsaurus (https://cellosaurus.org)
(c) ATCC (https://atcc.org)

(iv) Literature annotation
  - PubChem (https://pubmed.ncbi.nlm.nih.gov)/
    NCBI (https://ncbi.nlm.nih.gov)

### Visibility

Several additional measures were taken to make the dataset and its content visible to the scientific community; particularly,

(i) the HD_BPMDS is deposited and freely available under the very same file name (HD_BPMDS_Version_3_October_2023) on three independent repositories, allowing for its access from anywhere in the world, specifically
  - https://zenodo.org [12]
  - https://panabc.info [13]
  - https://OSF.io [14]
(ii) The DOIs generated by zenodo and OSI.io are cited within this manuscript, and thus, will be linked to benchmark databases, such as NCBI or GoogleScholar allowing researchers to easily find the dataset and original literature
(iii) The HD_BPMDS is freely available without restrictions (e.g., password, paywall, etc*.*)
(iv) The HD_BPMDS is provided as both xlsx. and csv format, enabling its interoperability
(v) the chemical formulae and molecular substructures are primarily represented as SMILES codes that are readable by any cheminformatics toolkit
(vi) The compound as well as substructure identifiers were allocated under consideration of our previous works [6, 15], promoting the visibility of already established compound/substructure labels and reducing confusion by multiple identifiers
(vii) This manuscript as well as the HD_BPMDS are published under a BY-CC 4.0 license, enabling anyone to access, analyze, process, and re-organize the given data for non-commercial and commercial purposes under referral to the originators.

## Limitation

### Outline

The HD_BPMDS is not limited with respect to its annotation and visibility providing an inclusive insight into the currently known landscape of HD-targeting agents with prospect of expansion into unknown chemical, bioactivity, or target space. However, general limitations are given by

(i) the limited number of studies demonstrating in vitro and/or in vivo efficacy in HD models
(ii) the limited number of clinical trials and case studies with drug candidates against HD
(iii) The exclusion of studies on named HD targets that did not link their successful findings into actual HD models
(iv) the very limited information of binding affinities to HD targets and used doses due to the limited number of studies as well as non-standardized and non-harmonized assay and treatment procedures

The lack of studies expanding the chemical, bioactivity, and/or compound space is the major impediment of the HD_BPMDS. In vitro assays with potential HD drug targets can indeed be found on PubMed (https://pubmed.ncbi.nlm.nih.gov), however, most studies did not implement complementary assessment with HD models involving HD pathology or biomarkers (e.g., muHTT-mediated toxicity). The obvious gap between pre-clinical success and actual clinical use prompted us to exclude all studies not immediately demonstrating efficacy of the tested compounds in at least one HD model, as inclusion of agents without this ability would have inevitably led to their annotation with successful pre-clinical efficacy. Reports including in vivo models, on the other hand, are genuinely not largely presented in the literature landscape as in vivo experiments are costly and adhere to regulatory constraints (e.g., ethic guidelines, animal welfare, maintenance costs, personnel education and training, etc*.*).

One major result of the lack of in vitro and in vivo reports is the lack of affinity and bioactivity values (e.g., k_i_, IC_50_, EC_50_, etc*.*). No standardized or harmonized assay procedures exist in the field of cell-based assays, and the degree of complexity of the given and variable parameters is even advanced regarding in vivo experiments. These assay- and experiment-specific variations limit the overall comparability of bioactivity data. Diverse data can indeed be compared and used as demonstrated in our previous study about the ABC_BPMDS [6]. However, in the case of the ABC_BPMDS, the assay variations concerned one target (super)family only, while the HD_BPMDS includes various targets and even (to a small extent) pathways. These aspects prompted us to not include bioactivity data into the HD_BPMDS at this stage. Additionally, the limitation in reports with an in parallel very diverse target landscape provided also very diverse assessment platforms that can be compared to one another to very limited extent only.

### Applicability domain

Nevertheless, the inclusion of 261 unique molecular substructures in a clear binary pattern distribution scheme enriches the dataset with molecular-structural information that allows for the identification of novel molecular entities by screening of chemical space. Recently, a similar fragment-based approach with a much smaller set of descriptors resulted in the successful identification of hit molecules [27], giving a positive prospect on the future use of the HD_BPMDS.

In order to validate the HD_BPMDS with respect to its applicability domain, we generated three distinct fingerprints from the binary code, namely (i) a target-specific fingerprint of 13 histone deacetylases-(HDACs)-focusing molecules (Additional file 1: Table S1); (ii) a target-specific fingerprint of 16 heat shock proteins-(HSPs)-focusing molecules (Additional file 2: Table S2); and (iii) a negative fingerprint obtained from the entire dataset taking the least occurring substructures (only once in 429 compounds of the dataset; Additional file 3: Table S3) into account.

Both target-specific fingerprints, which consisted of the 75% most occurring substructures within the respective sets of compounds, were searched for the 17,350 and 6035 reported and unique HDAC and HSP modulators, respectively, curated from the ChMEBL database (https://ebi.ac.uk/chembl). Applying these substructures cumulatively, the HDACs-specific fingerprint was able to return 1191 molecules from the ChEMBL-listed HDAC molecles (6.86%), while the HSPs-specific fingerprint returned 1448 molecules (24.0%). Interestingly, only 4 and 3 of the found 1191 and 1448 HDACs- and HSPs-targeting agents, respectively, were part of the initial sets of compounds both the HDACs- and HSPs-specific fingerprints were deduced from. This indicates that 99.7% and 99.8% of the molecules fell outside the molecular-structural constraints of the HD-BPMDS, which reflects the extensive scope of applicability of the HD-BPMDS.

With respect to the negative fingerprint, the 39 least occurring substructures of the HD_BPMDS were searched for in both the 17,350 HDACs- and 6035 HSPs-targeting compounds as available from ChEMBL. Strikingly, each of the substructures massively reduced the number of compounds. On average, the negative fingerprint substructure were found in 41 and 18 HDACs- and HSPs-targeting molecules only (0.236% and 0.298%, respectively.

Although these numbers must be handled with care as the respective fingerprints were target subtype-unspecific (general HDACs/HSPs modulators), and particularly the positive fingerprint contained rather unspecific substructures with minor substructural variation, it can generally be stated that target class- (and pathway-)specific fingerprints derived from the HD_BPMDS have an extended applicability domain beyond the molecular-structural limitations of the HD_BPMDS and may be used specifically to shape the (poly)pharmacological profile of future therapeutics. In our earlier work we demonstrated that pattern analysis allowed for an accurate prediction of biological hit compounds at a fairly low number of tested compounds [7–9]. The datasets underlaying these predictions contained > 1000 compounds each at the time of the respective study [6–9]. Interestingly, the HD_BPMDS showed that even much smaller input data (429 compounds) was sufficient to provide a large opportunity space beyond the molecular-structural limitation as an immediate result of the low number of compounds.

### Physicochemical and molecular–structural validation

**Fig. 1:**
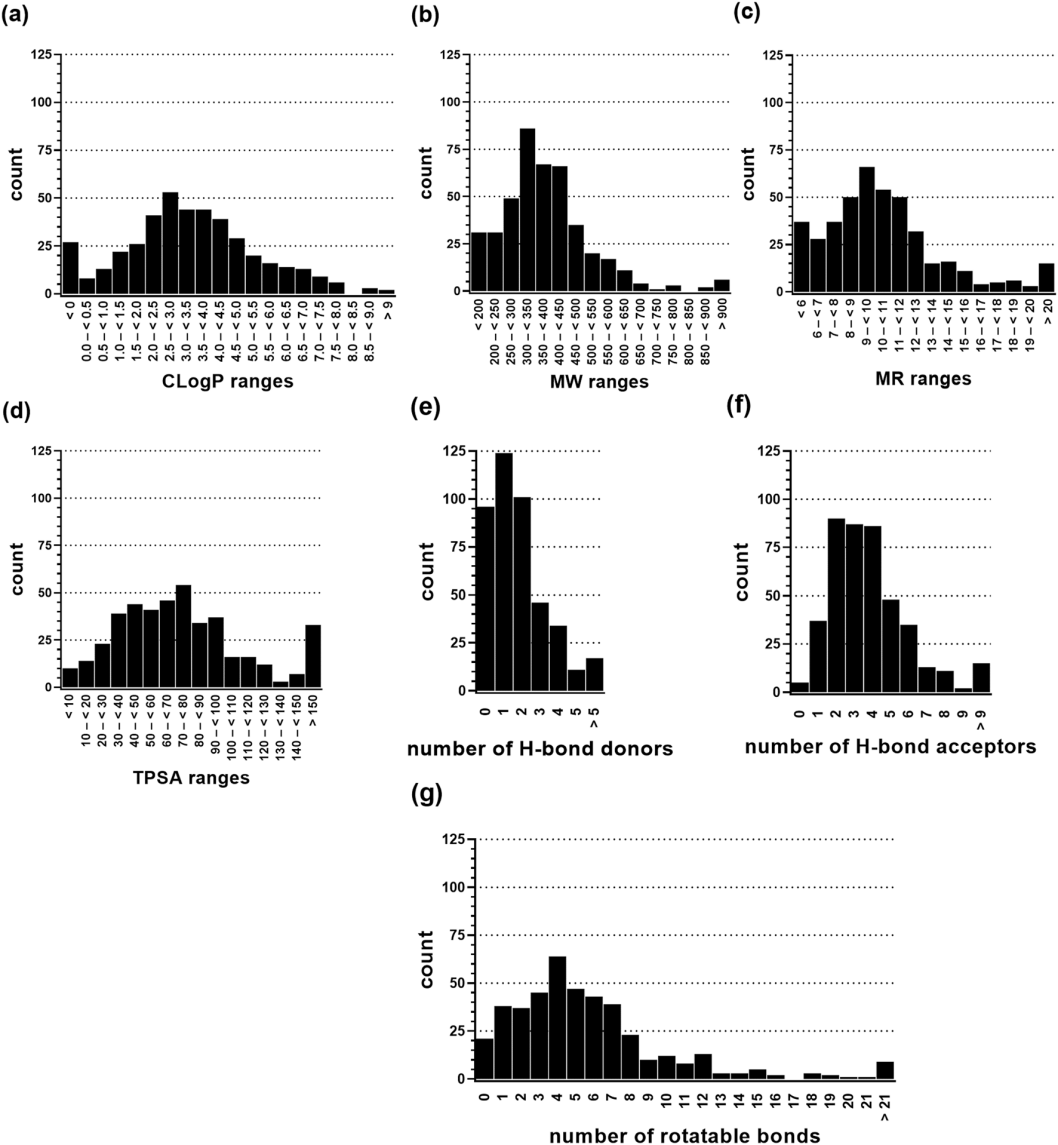
Distribution of physicochemical and molecular-structural attributes of the 429 molecules of the HD BPMDS as determined by MOE version 2019.01. **a** Calculated octanol–water partition coefficient (CLogP). **b** Molecular weight (MW). **c** Molar refractivity (MR). **d** Topological polar surface area (TPSA). **e** H bond donors. **f **H-bond acceptors. **g** Rotatable bonds

A balanced distribution of physicochemical (e.g., CLogP, MW, MR, and TPSA) and molecular-structural (e.g., H-bond donors/acceptors, rotatable bonds) parameters contributes to the validity of datasets. Figure 1 visualizes the analysis of the entire 429 molecules of the HD_BPMDS toward the above named features, which were all distributed in a gaussian manner. Analyses of subsets, i.e., HDACs-, HSPs-, solute carriers-(SLCs)-, ion channels-(ICs)-, (tyrosine) kinases-[(T)Ks]-, and sigma receptors-(σRs)-focusing molecules (Additional file 4: Figs. S1–6), compound categories (i–iii) molecules (Additional file 4: Figs. S7–9), target categories (i–v)-focusing molecules (Additional file 4: Figs. S14), agonists/activators and antagonists/inhibitors (Additional file 4: Figs. S15–16), as well as early and late drug development stage molecules (Additional file 4: Figs. S17–18) supported these findings.

Generally, the individual analyses revealed either equal or Gaussian distributions; the cases in which this was less/not observed were mainly subsets with very low numbers of analyzed compounds. The median and mean values of the entire dataset as well as the subsets are well-aligned underlining the equal distribution the analyzed attributes (Additional file 5: Table S4).

## Conclusions

The HD_BPMDS provides inclusive molecular-structural knowledge with an applicability domain beyond its limitations regarding compound-, bioactivity-, and target-related constraints. Despite the rather low number of compounds, particularly with respect to the addressed target classes, fingerprints derived from these target classes can be used for future virtual screening or rational drug design approaches to shape the (poly)pharmacological profile of HD- (or non-HD-)targeting drugs of the future. In addition, the present work demonstrated also the superiority of pattern analysis in terms of ‘negative fingerprints’ that can be used to make certain pharmacological effects in drug design approaches more unlikely. On the other hand, frequently occurring substructures like pyridine (18.8% of HD_BPMDS compounds), pyrimidine (7.5%), or thiazole (7.0%) in combination with (hetero)aliphatic patterns could represent the backbone for future drug and target repurposing strategies for the development of novel HD-targeting agents, particularly addressing the uncharted territory of target space.

### Supplementary Information

**Additional file 1.** HDACs-specific fingerprint.

**Additional file 2.** HSPs-specific fingerprint.

**Additional file 3.** HD-specific negative fingerprint.

**Additional file 4.** Physicochemical and molecular-structural validation (visualized graphics).

**Additional file 5.** Physicochemical and molecular-structural validation (numeric values).

## Data Availability

The dataset (version 3) is freely available at: (i) zenodo (https://doi.org/10.5281/zenodo.8363783)) [12]; (ii) PANABC.info (http://www.panabc.info) [13]; (iii) OSI.io (http://www.doi.org/10.17605/OSF.IO/EJVWY) [14]. The original dataset (version 1) is freely available at (i) zenodo (https://doi.org/10.5281/zenodo.7854956).

## Abbreviations

ABC transporters: ATP-binding cassette transporters
AI: Artificial intelligence
ATCC: American Type Culture Collection
ATP: Adenosine-triphosphate
CLogP: Calculated octanol–water partition coefficient
CLogS: Calculated solubility
C@PA: Computer-aided pattern analysis
DNA: Deoxyribonucleic acid
DOI: Digital object identifier
EC_50_: Half-maximal effect concentration
[H]: Defined hydrogen
H-bond: Hydrogen bond
HD: Huntington’s disease
HD_BPMDS: HD-focusing binary pattern MDS
HDACs: Histone deacetylases
HSPs: Heat shock proteins
HTS: High-throughput screening
HTT: Huntingtin
IC_50_: Half-maximal inhibition concentration
IUPHAR: International Union of Basic and Clinical Pharmacology
k_i_: Affinity constant
MDSs: Multitarget datasets
ML: Machine learning
MR: Molar refractivity
muHTT: Mutated HTT
MW: Molecular weight
NNs: Neural networks
RNA: Ribonucleic acid
SMILES: Simplified molecular input line entry specification
TPSA: Topological polar surface area
