# Supplementary material for: HD_BPMDS: a curated binary pattern multitarget dataset of Huntington’s disease–targeting agents
Source: J Cheminform. 2023 Nov 17;15:109. doi: 10.1186/s13321-023-00775-z (PMC10655317; doi:10.1186/s13321-023-00775-z)
Supplement: Supplementary file 4 — Additional file 4. Physicochemical and molecular-structural validation (visualized graphics). [file 13321_2023_775_MOESM4_ESM.pdf]

**HD_BPMDS: a curated binary pattern multitarget dataset of Huntington's disease–targeting agents**

*Sven Marcel Stefan [1,2,3], Jens Pahnke [1,2,4,5], Vigneshwaran Namasivayam [2,6,*]*

[1] Department of Pathology, Section of Neuropathology, Translational Neurodegeneration Research and Neuropathology Lab (www.pahnkelab.eu), University of Oslo and Oslo University Hospital, Sognsvannsveien 20, 0372 Oslo, Norway

[2] Drug Development and Chemical Biology, Lübeck Institute of Experimental Dermatology (LIED), University of Lübeck and University Medical Center Schleswig-Holstein, Ratzeburger Allee 160, 23538 Lübeck, Germany

[3] School of Medical Sciences, Faculty of Medicine and Health, The University of Sydney, Camperdown NSW 2006, Australia

[4] Department of Pharmacology, Faculty of Medicine, University of Latvia, Jelgavas iela 4, 1004 Rīga, Latvia

[5] Department of Neurobiology, The Georg S. Wise Faculty of Life Sciences, Tel Aviv University, 6997801, Tel Aviv, Israel

[6] Department of Pharmaceutical and Cellbiological Chemistry, Pharmaceutical Institute, University of Bonn, An der Immenburg 4, 53121 Bonn, Germany

**Additional File 4**

***Histone deacetylases-(HDACs)-focusing molecules:***

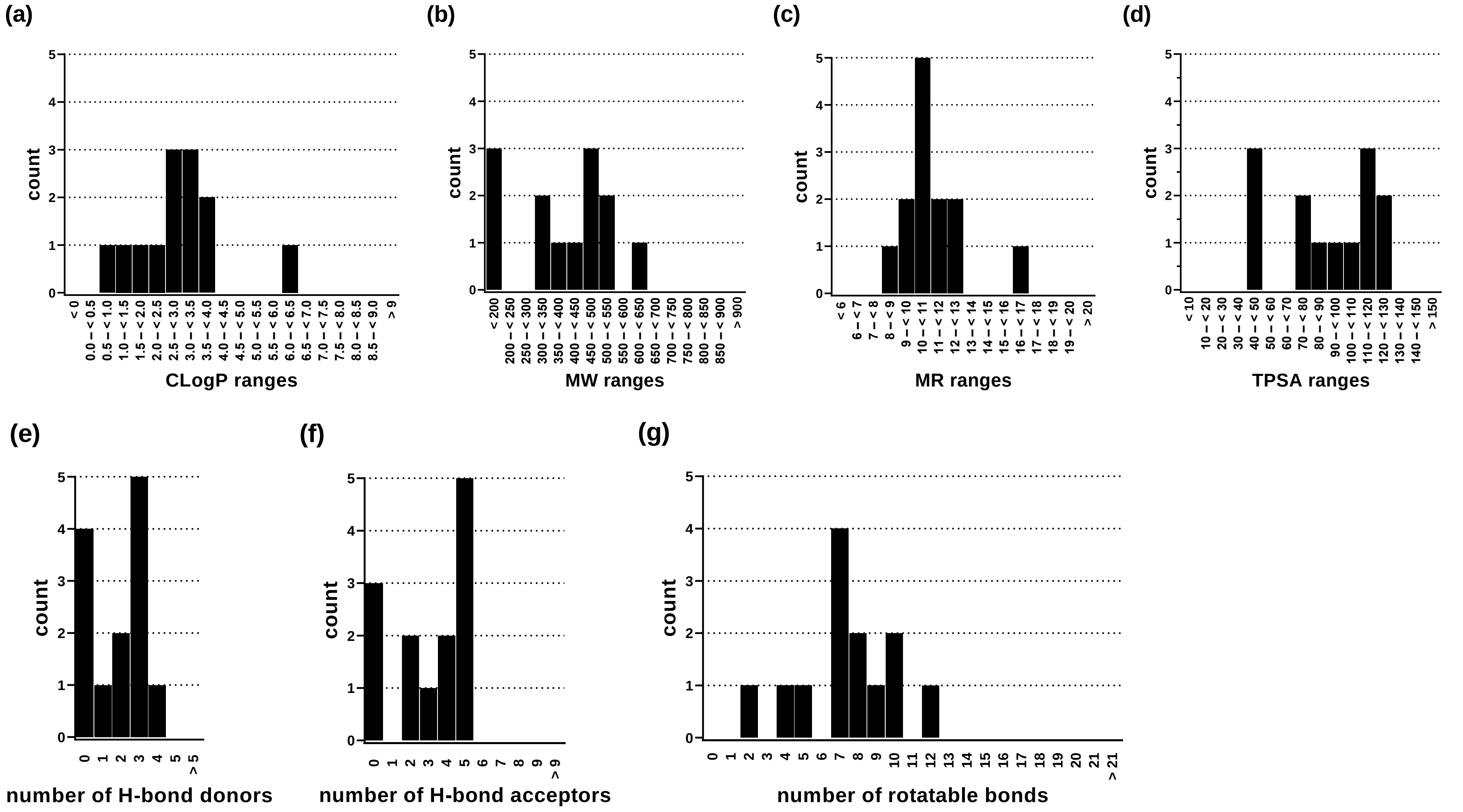


**Fig. S1** Distribution of physicochemical and molecular-structural attributes of the 13 HDACs-focusing molecules of the HD_BPMDS as determined by MOE version 2019.01. **a** Calculated octanol-water partition coefficient (CLogP). **b** Molecular weight (MW)**.** **c** Molar refractivity (MR). **d** Topological polar surface area (TPSA). **e** Hydrogen-(H)-bond donors. **F** H-bond acceptors. **g** Rotatable bonds.

***Heat shock proteins-(HSPs)-focusing molecules*:**

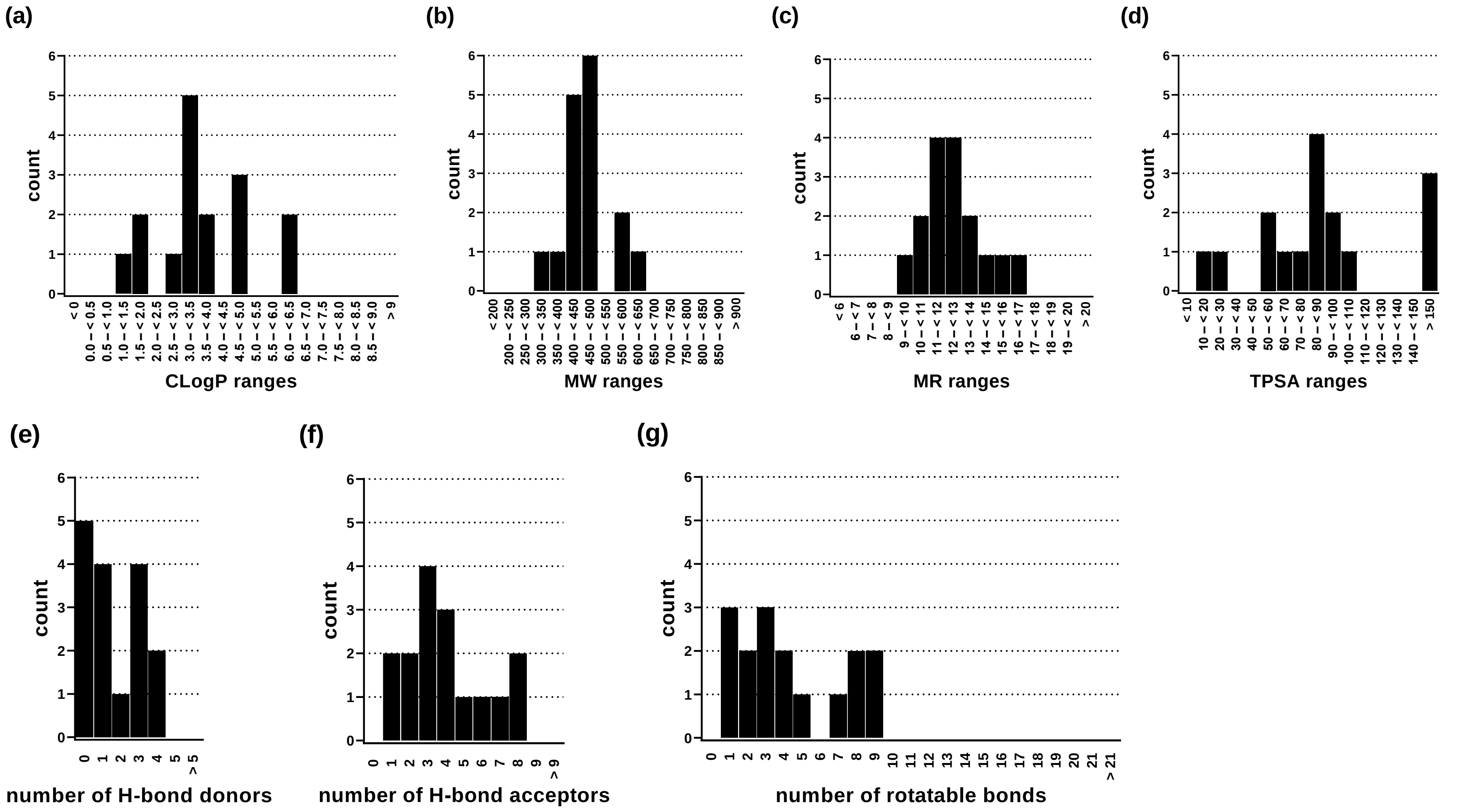


**Fig. S2** Distribution of physicochemical and molecular-structural attributes of the 16 HSPs-focusing molecules of the HD_BPMDS as determined by MOE version 2019.01. **a** Calculated octanol-water partition coefficient (CLogP). **b** Molecular weight (MW)**. c** Molar refractivity (MR). **d** Topological polar surface area (TPSA). **e** H-bond donors. **f** H-bond acceptors. **g** Rotatable bonds.

***Solute carriers-(SLCs)-focusing molecules*:**

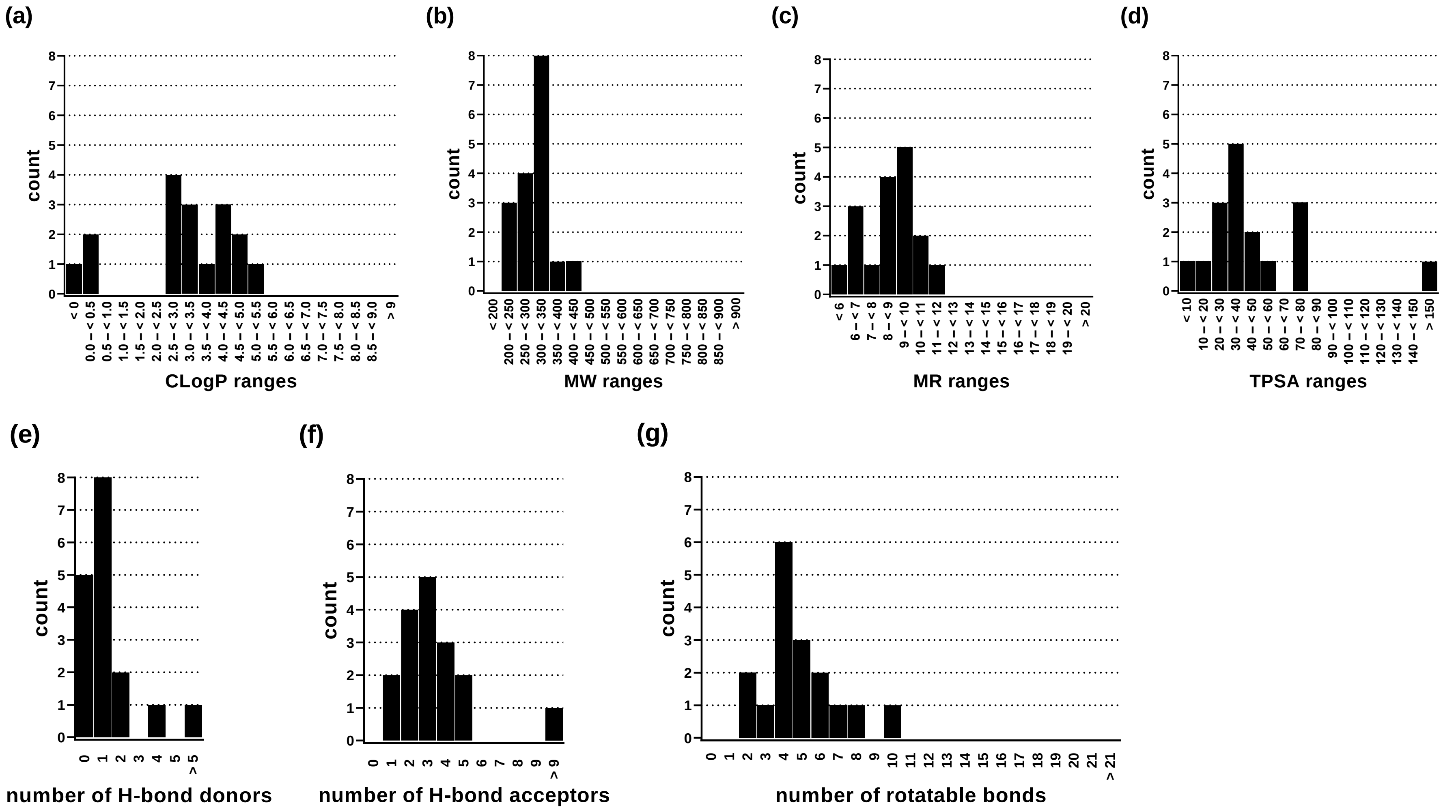


**Fig. S3** Distribution of physicochemical and molecular-structural attributes of the 17 SLCs-focusing molecules of the HD_BPMDS as determined by MOE version 2019.01. **a** Calculated octanol-water partition coefficient (CLogP). **b** Molecular weight (MW)**. c** Molar refractivity (MR). **d** Topological polar surface area (TPSA). **e** H-bond donors. **f** H-bond acceptors. **g** Rotatable bonds.

***Ion channels-(ICs)-focusing molecules*:**

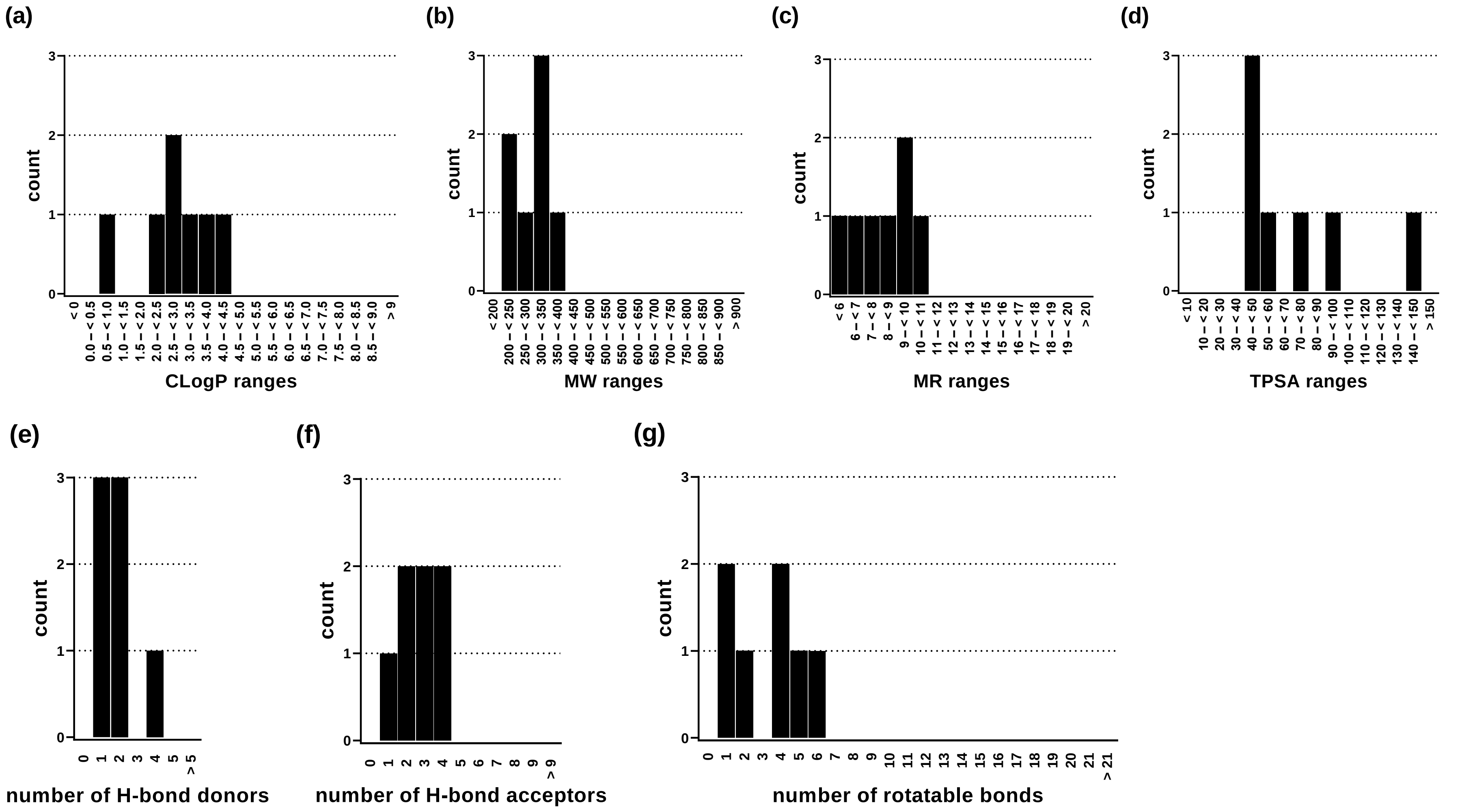


**Fig. S4** Distribution of physicochemical and molecular-structural attributes of the 7 ICs-focusing molecules of the HD_BPMDS as determined by MOE version 2019.01. **a** Calculated octanol-water partition coefficient (CLogP). **b** Molecular weight (MW)**. c** Molar refractivity (MR). **d** Topological polar surface area (TPSA). **e** H-bond donors. **f** H-bond acceptors. **g** Rotatable bonds.

***(Tyrosine) Kinases-[(T)Ks]-focusing molecules:***

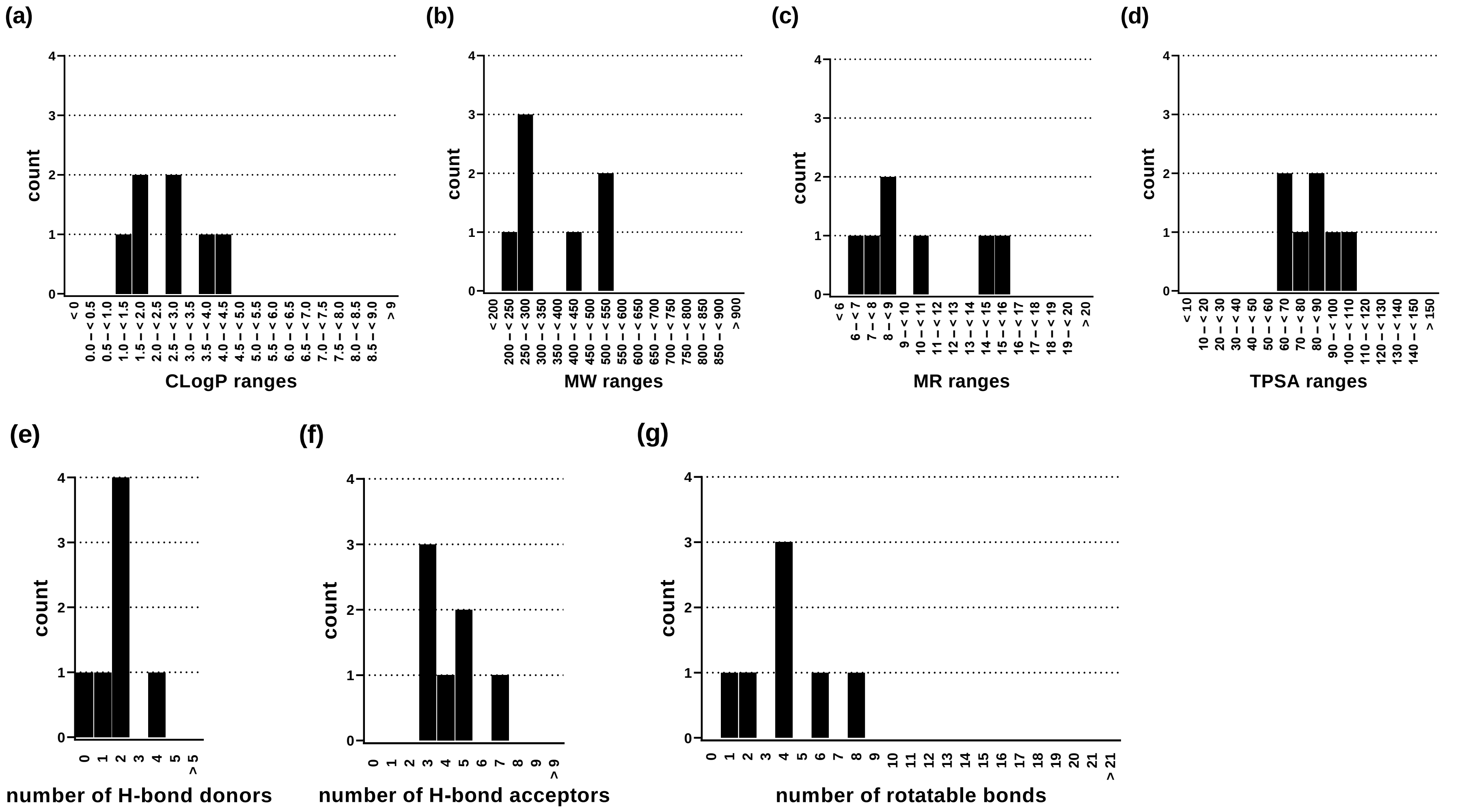


**Fig. S5** Distribution of physicochemical and molecular-structural attributes of the 7 (T)Ks-focusing molecules of the HD_BPMDS as determined by MOE version 2019.01. **a** Calculated octanol-water partition coefficient (CLogP). **b** Molecular weight (MW)**. c** Molar refractivity (MR). **d** Topological polar surface area (TPSA). **e** H-bond donors. **f** H-bond acceptors. **g** Rotatable bonds.

***Sigma receptors-(σRs)-focusing molecules:***

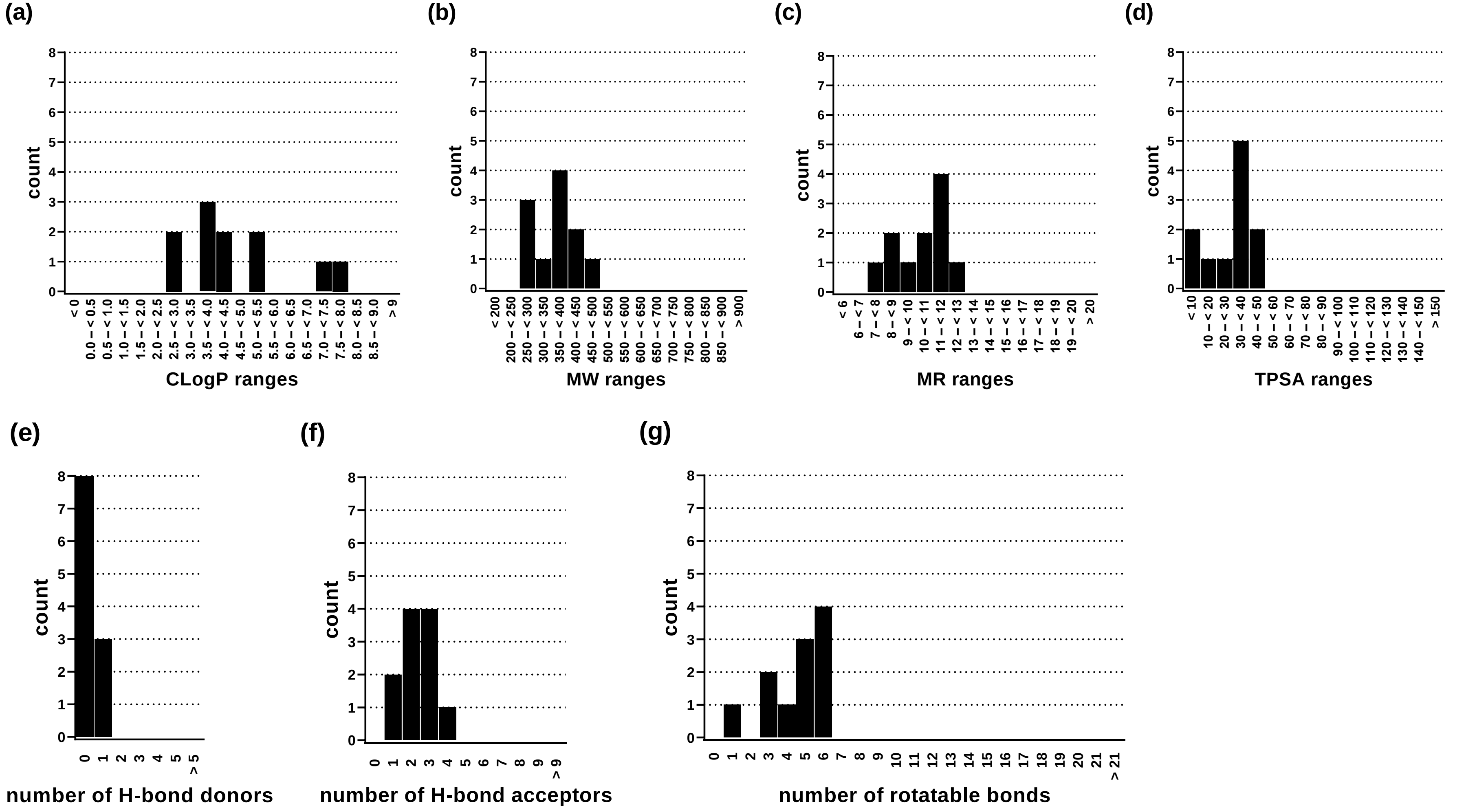


**Fig. S6** Distribution of physicochemical and molecular-structural attributes of the 11 σRs-focusing molecules of the HD_BPMDS as determined by MOE version 2019.01. **a** Calculated octanol-water partition coefficient (CLogP). **b** Molecular weight (MW)**. c** Molar refractivity (MR). **d** Topological polar surface area (TPSA). **e** H-bond donors. **f** H-bond acceptors. **g** Rotatable bonds.

***<u>Compound category (i) molecules (‘Pharmaceutical Drug / Diagnostic’):</u>***

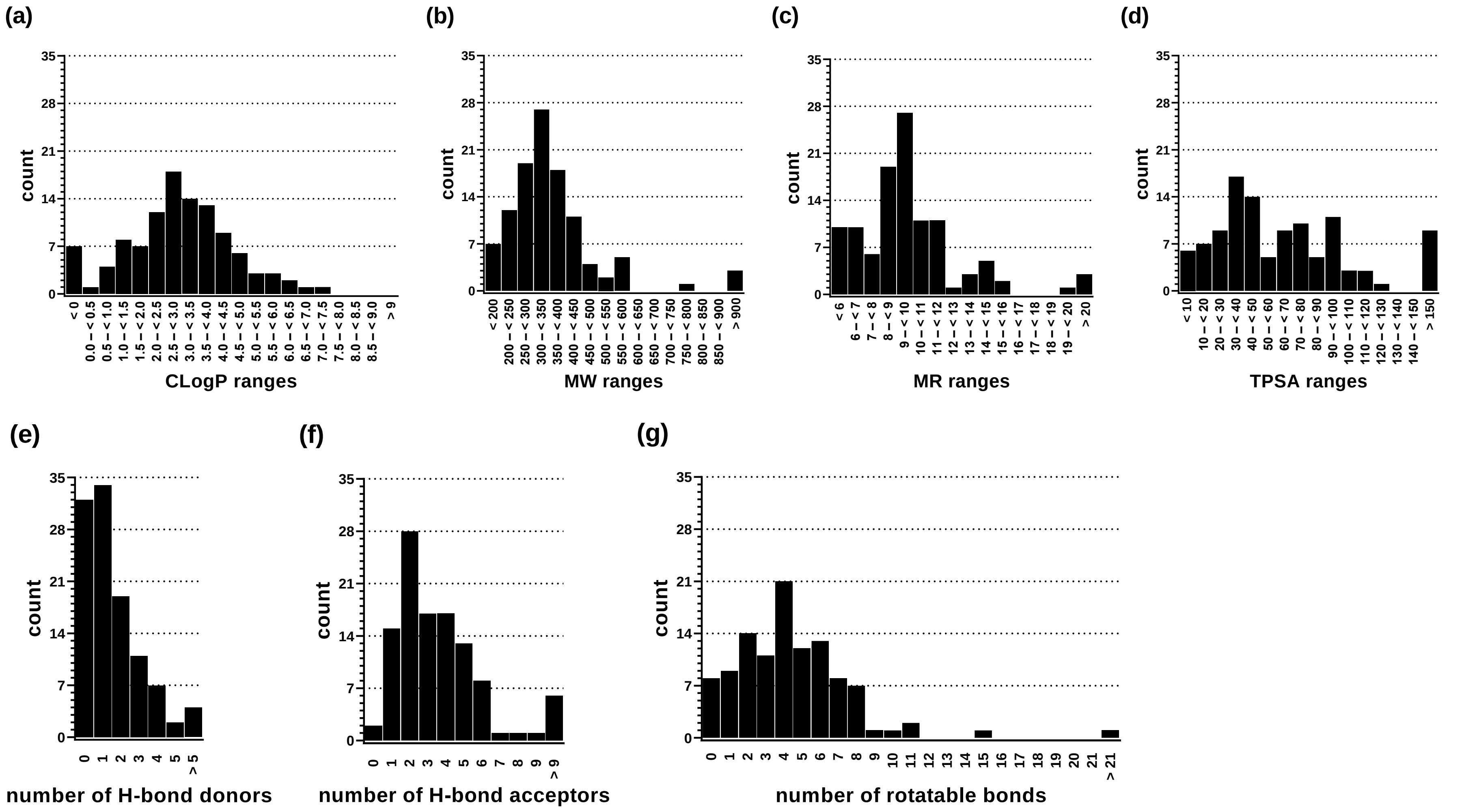


**Fig. S7** Distribution of physicochemical and molecular-structural attributes of the 109 compound category (i) molecules (‘Pharmaceutical Drug / Diagnostic’) of the HD_BPMDS as determined by MOE version 2019.01. **a** Calculated octanol-water partition coefficient (CLogP). **b** Molecular weight (MW)**. c** Molar refractivity (MR). **d** Topological polar surface area (TPSA). **e** H-bond donors. **f** H-bond acceptors. **g** Rotatable bonds.

***Compound category (ii) molecules (‘Drug-like Compound / Chemical Substance’):***

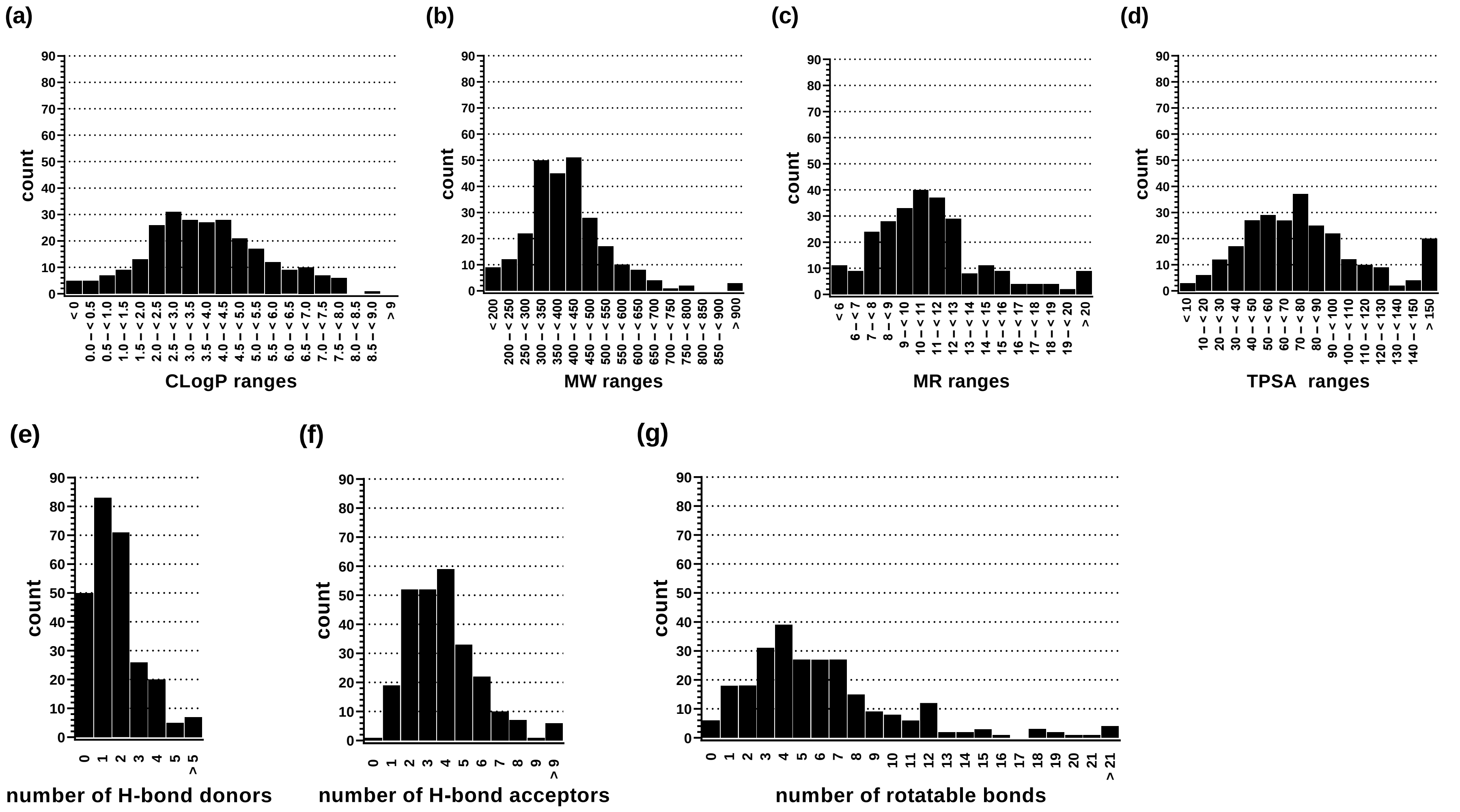


**Fig. S8** Distribution of physicochemical and molecular-structural attributes of the 262 compound category (ii) molecules (‘Drug-like Compound / Chemical Substance’) of the HD_BPMDS as determined by MOE version 2019.01. **a** Calculated octanol-water partition coefficient (CLogP). **b** Molecular weight (MW)**.** **c** Molar refractivity (MR). **d** Topological polar surface area (TPSA). **e** H-bond donors. **f** H-bond acceptors. **g** Rotatable bonds.

***Compound category (iii) molecules (‘Nutrient / Metabolite’):***

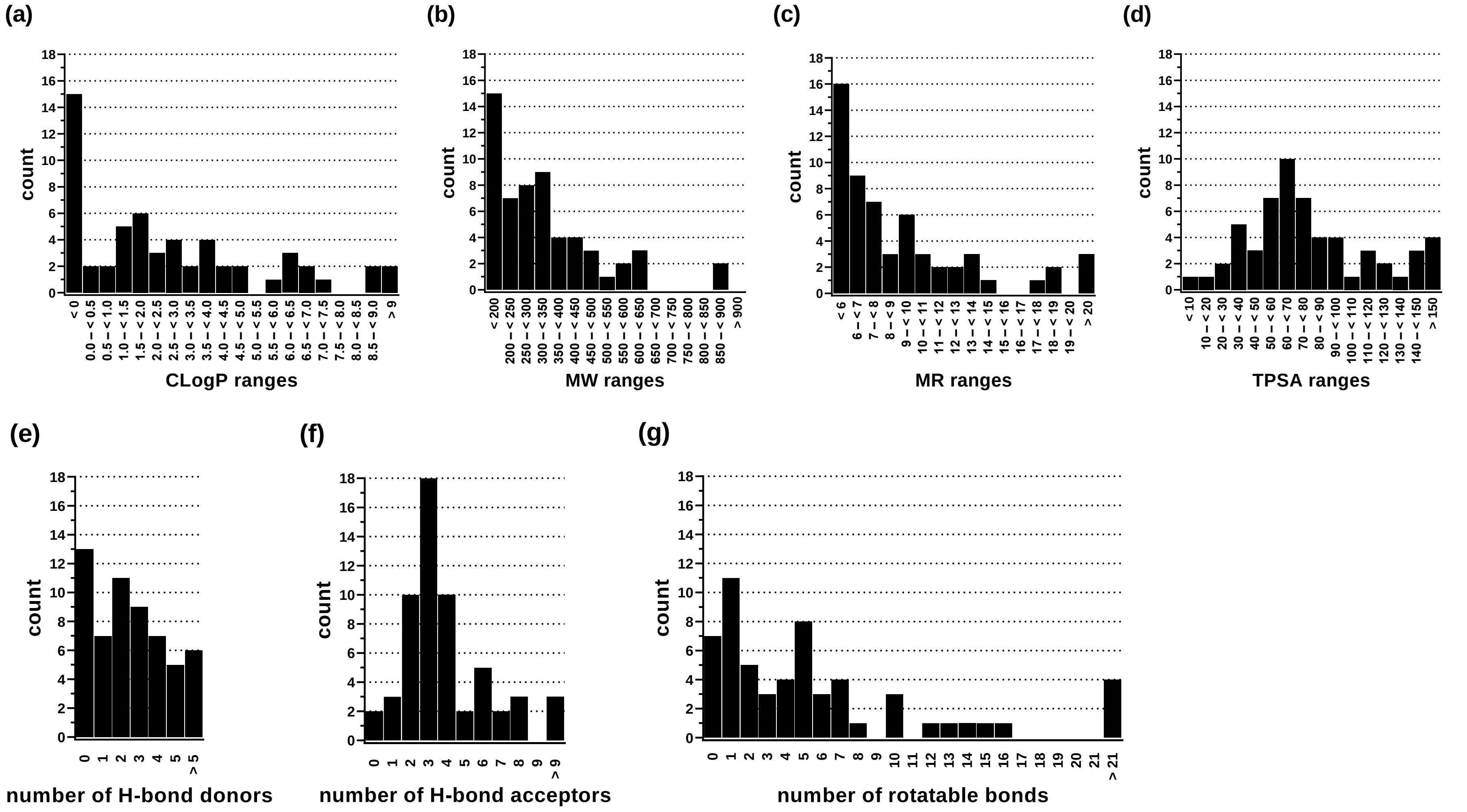


**Fig. S9** Distribution of physicochemical and molecular-structural attributes of the 58 compound category (iii) molecules (‘Nutrient / Metabolite’) of the HD_BPMDS as determined by MOE version 2019.01. **a** Calculated octanol-water partition coefficient (CLogP). **b** Molecular weight (MW)**. c** Molar refractivity (MR). **d** Topological polar surface area (TPSA). **e** H-bond donors. **f** H-bond acceptors. **g** Rotatable bonds.

***Target category (i)-focusing molecules ('Neurotransmitter Systems'):***

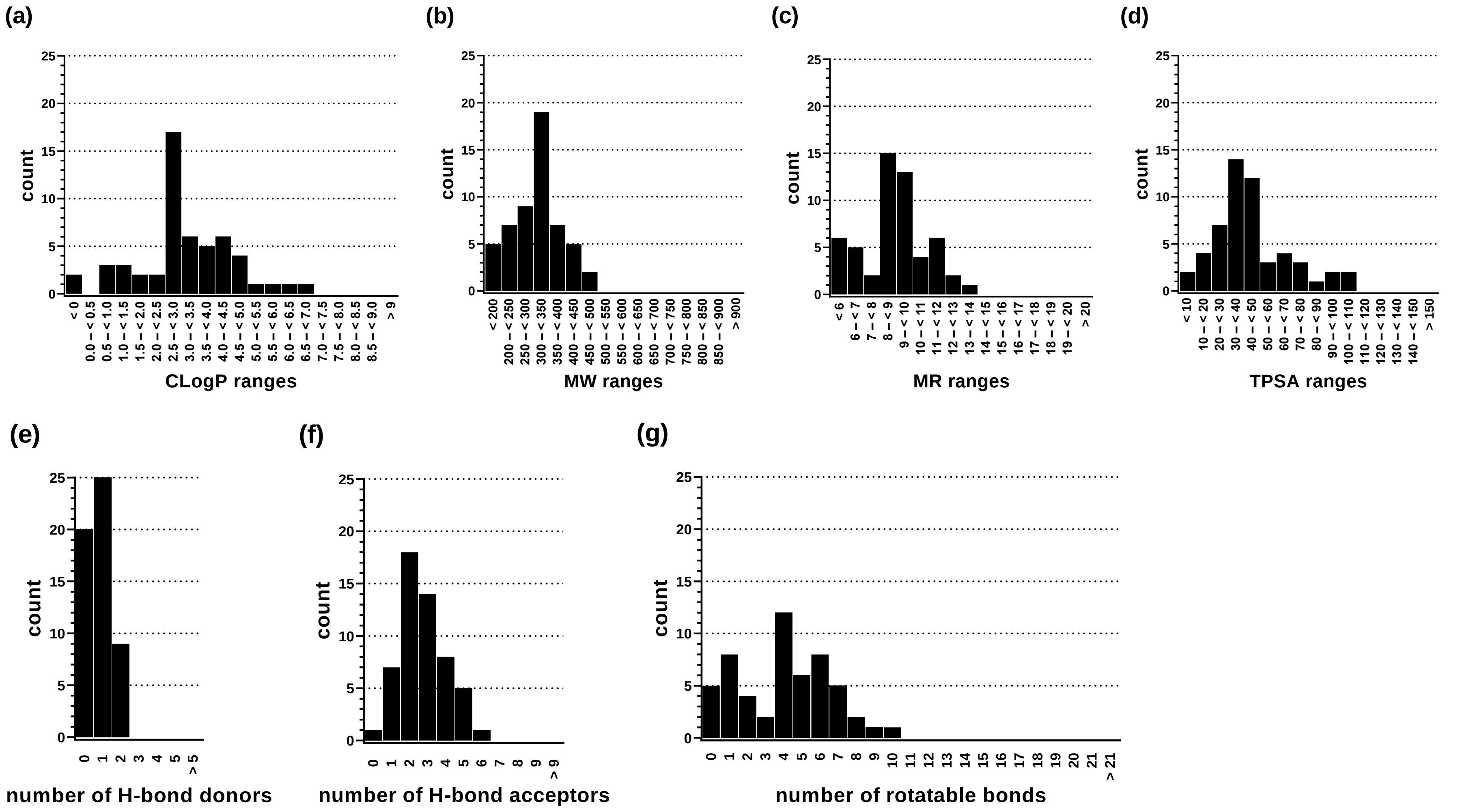


**Fig. S10** Distribution of physicochemical and molecular-structural attributes of the 54 target category (i)-focusing molecules ('Neurotransmitter Systems') of the HD_BPMDS as determined by MOE version 2019.01. **a** Calculated octanol-water partition coefficient (CLogP). **b** Molecular weight (MW)**. c** Molar refractivity (MR). **d** Topological polar surface area (TPSA). **e** H-bond donors. **f** H-bond acceptors. **g** Rotatable bonds.

***Target category (ii)-focusing molecules ('Mitochondrial Systems'):***

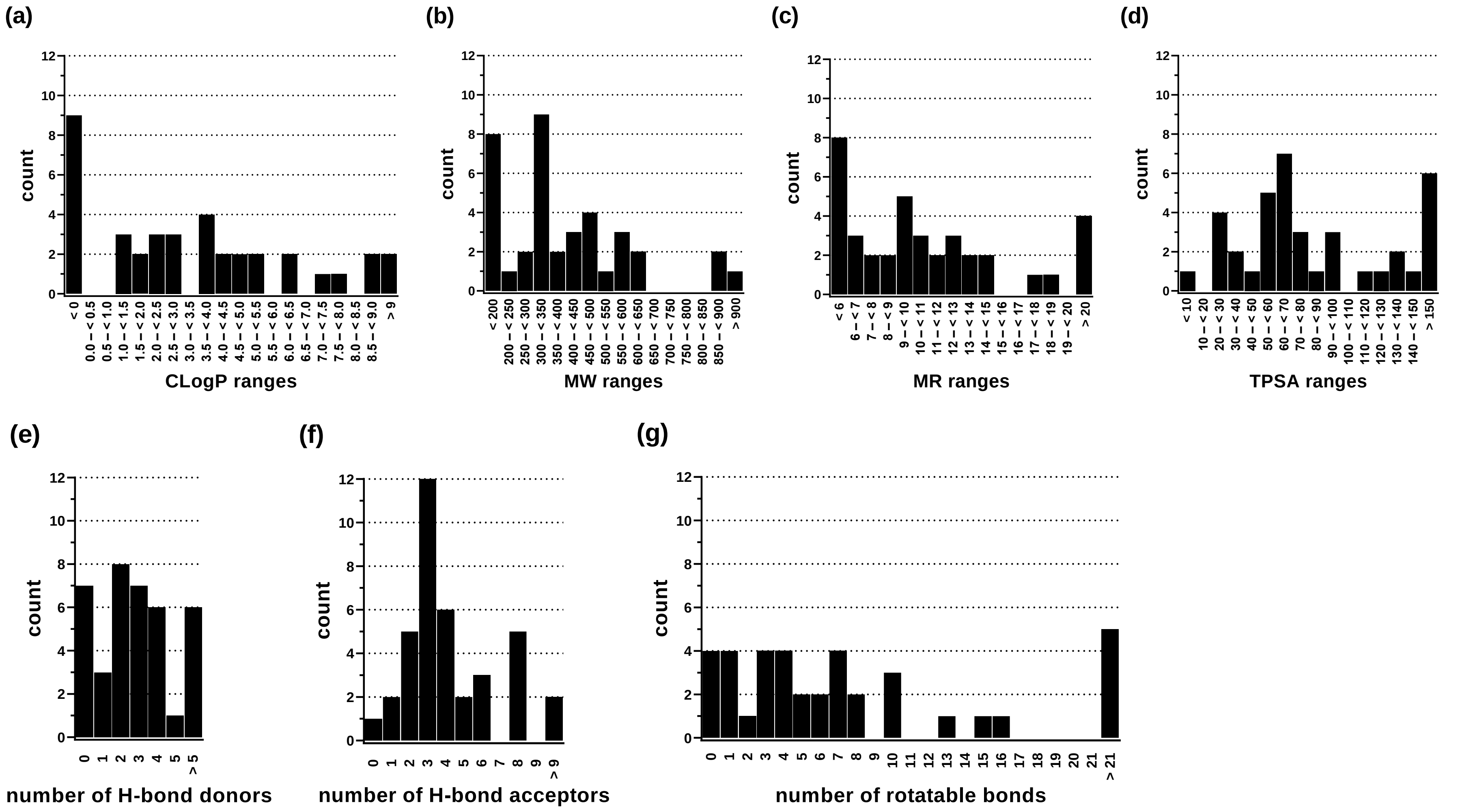


**Fig. S11** Distribution of physicochemical and molecular-structural attributes of the 38 target category (ii)-focusing molecules ('Mitochondrial Systems') of the HD_BPMDS as determined by MOE version 2019.01. **a** Calculated octanol-water partition coefficient (CLogP). **b** Molecular weight (MW)**. c** Molar refractivity (MR). **d** Topological polar surface area (TPSA). **e** H-bond donors. **f** H-bond acceptors. **g** Rotatable bonds.

***Target category (iii)-focusing molecules ('muHTT mRNA or DNA'):***

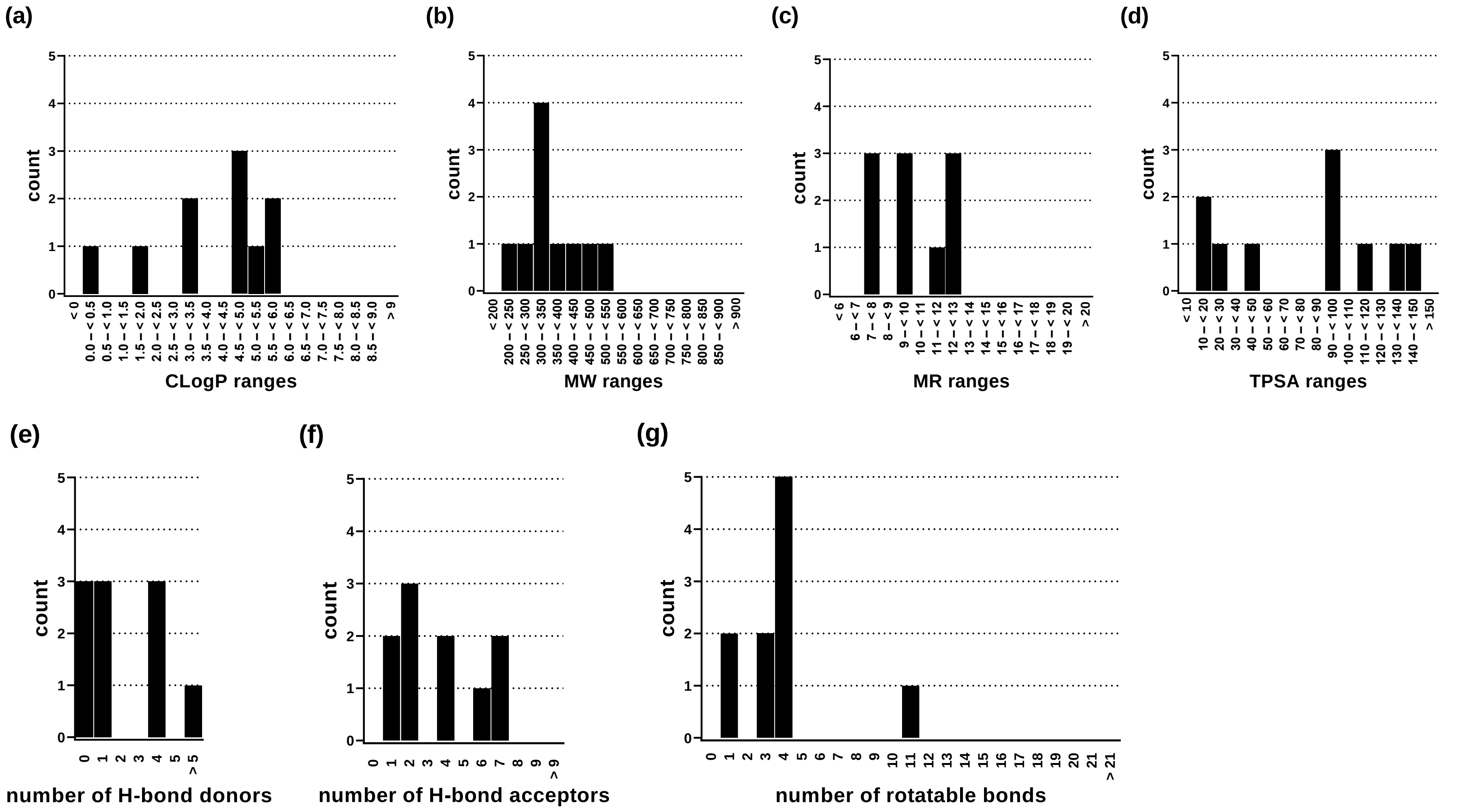


**Fig. S12** Distribution of physicochemical and molecular-structural attributes of the 10 target category (iii)-focusing molecules ('muHTT mRNA or DNA') of the HD_BPMDS as determined by MOE version 2019.01. **a** Calculated octanol-water partition coefficient (CLogP). **b** Molecular weight (MW)**.** **c** Molar refractivity (MR). **d** Topological polar surface area (TPSA). **e** H-bond donors. **f** H-bond acceptors. **g** Rotatable bonds.

***Target category (iv)-focusing molecules (‘muHTT Protein’):***

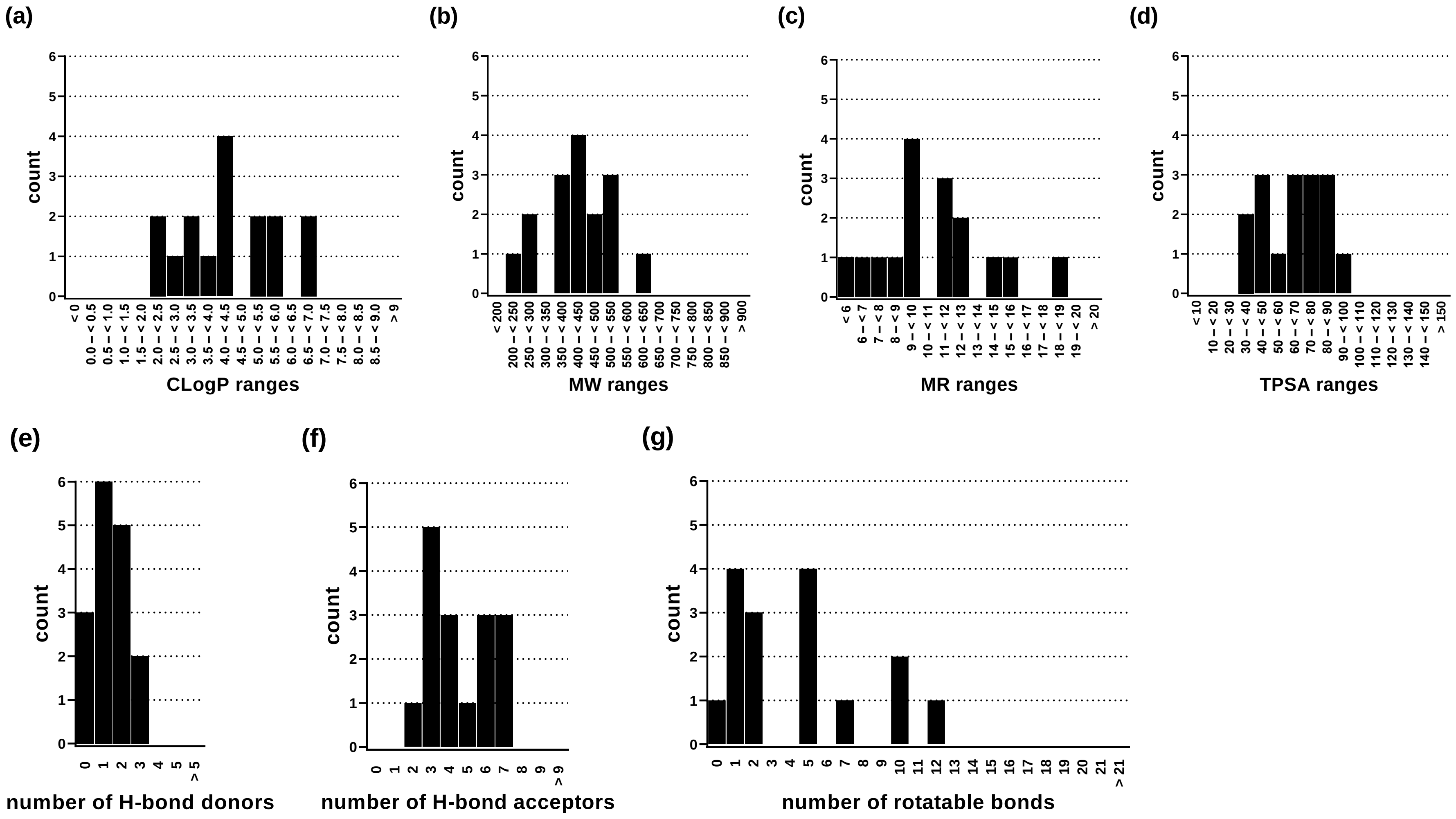


**Fig. S13** Distribution of physicochemical and molecular-structural attributes of the 16 target category (iv)-focusing molecules (‘muHTT Protein’) of the HD_BPMDS as determined by MOE version 2019.01. **a** Calculated octanol-water partition coefficient (CLogP). **b** Molecular weight (MW)**. c** Molar refractivity (MR). **d** Topological polar surface area (TPSA). **e** H-bond donors. **f** H-bond acceptors. **g** Rotatable bonds.

***Target category (v)-focusing molecules ('Novel Targets'):***

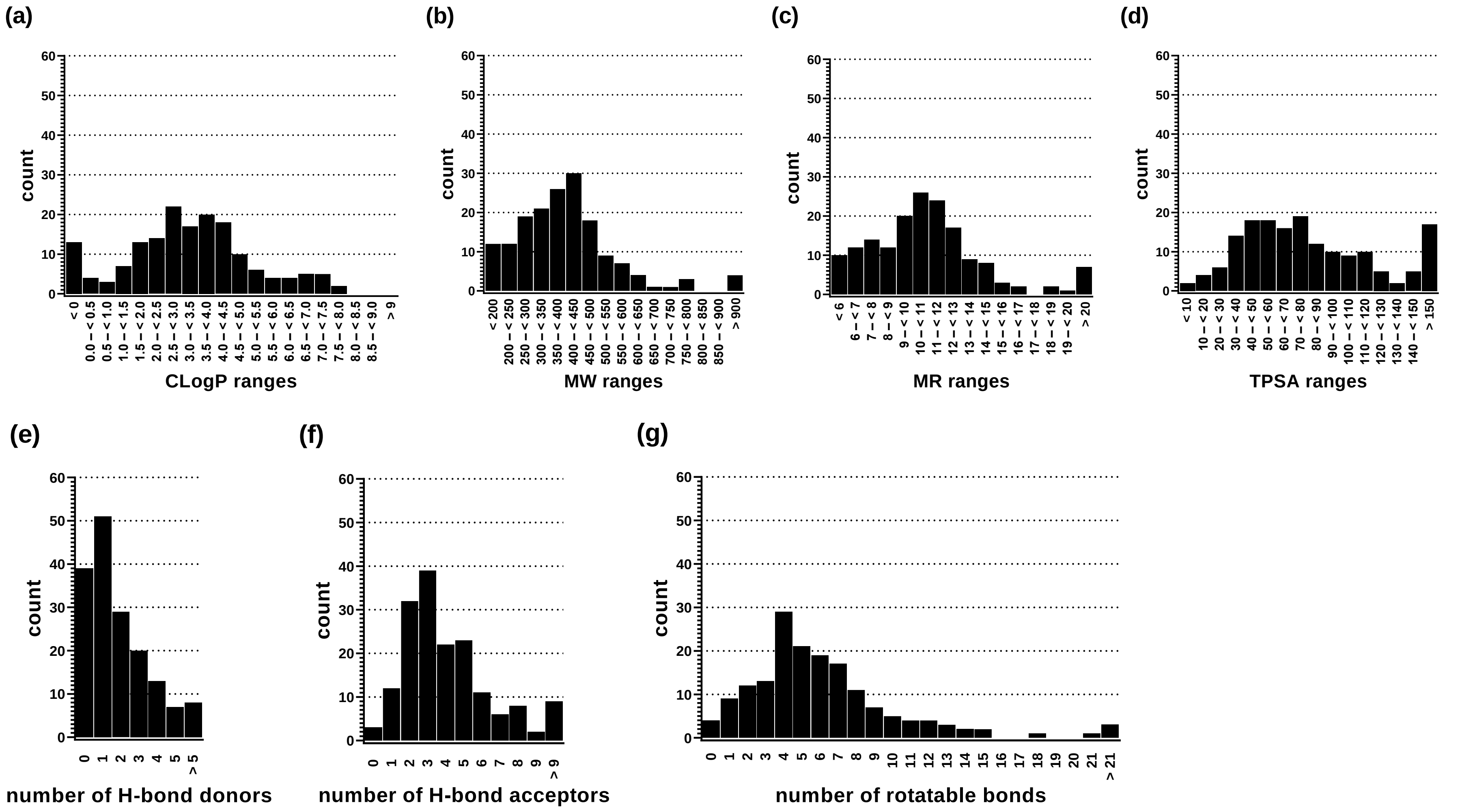


**Fig. S14** Distribution of physicochemical and molecular-structural attributes of the 167 target category (v)-focusing molecules ('Novel Targets') of the HD_BPMDS as determined by MOE version 2019.01. **a** Calculated octanol-water partition coefficient (CLogP). **b** Molecular weight (MW)**. c** Molar refractivity (MR). **d** Topological polar surface area (TPSA). **e** H-bond donors. **f** H-bond acceptors. **g** Rotatable bonds.

***Agonists / Activators:***

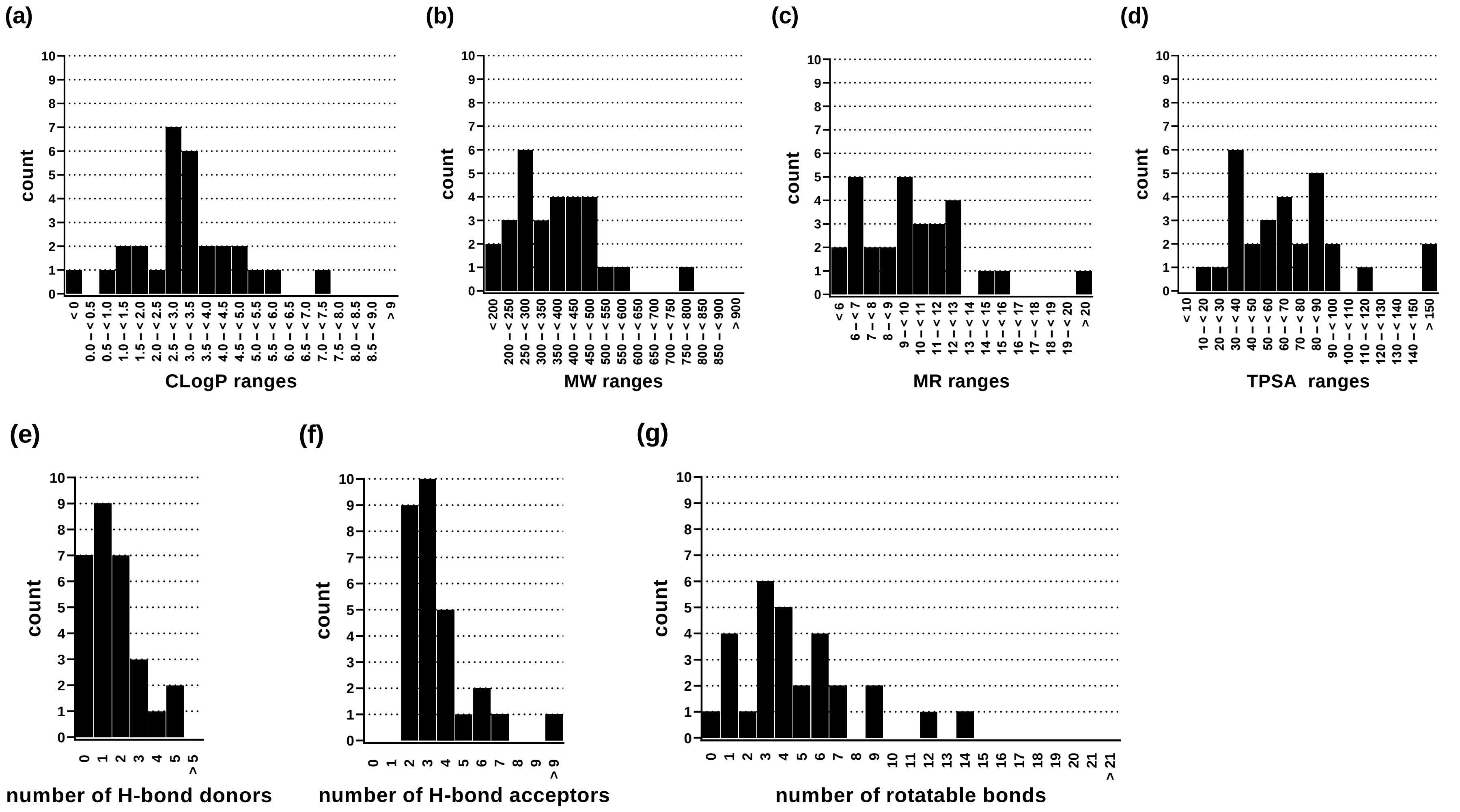


**Fig. S15** Distribution of physicochemical and molecular-structural attributes of the 29 agonists and activators of the HD_BPMDS as determined by MOE version 2019.01. **a** Calculated octanol-water partition coefficient (CLogP). **b** Molecular weight (MW)**. c** Molar refractivity (MR). **d** Topological polar surface area (TPSA). **e** H-bond donors. **f** H-bond acceptors. **g** Rotatable bonds.

***Antagonists / Inhibitors*:**

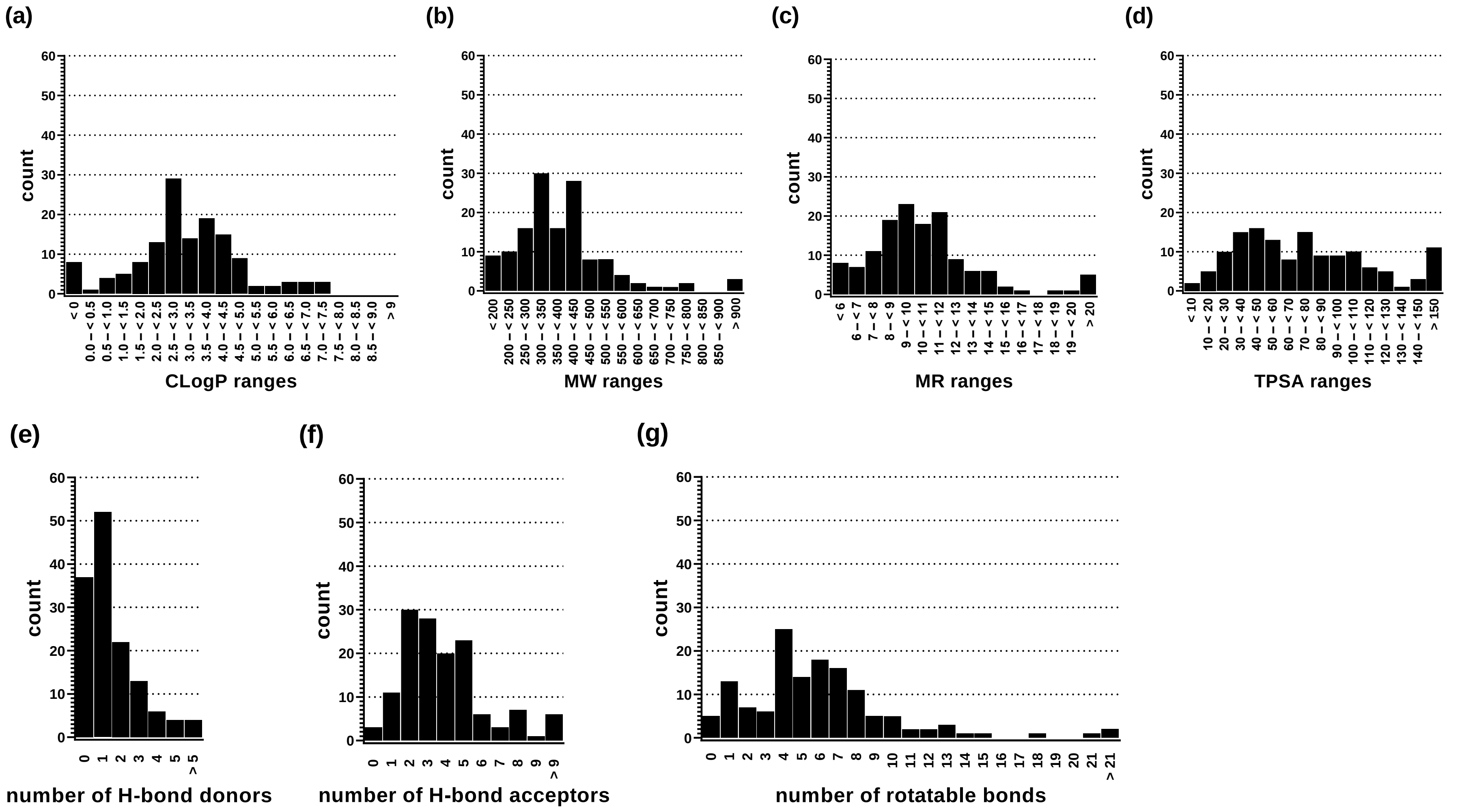


**Fig. S16** Distribution of physicochemical and molecular-structural attributes of the 138 antagonists and inhibitors of the HD_BPMDS as determined by MOE version 2019.01. **a** Calculated octanol-water partition coefficient (CLogP). **b** Molecular weight (MW)**. c** Molar refractivity (MR). **d** Topological polar surface area (TPSA). **e** H-bond donors. **f** H-bond acceptors. **g** Rotatable bonds.

***Early drug development stage molecules (in vitro):***

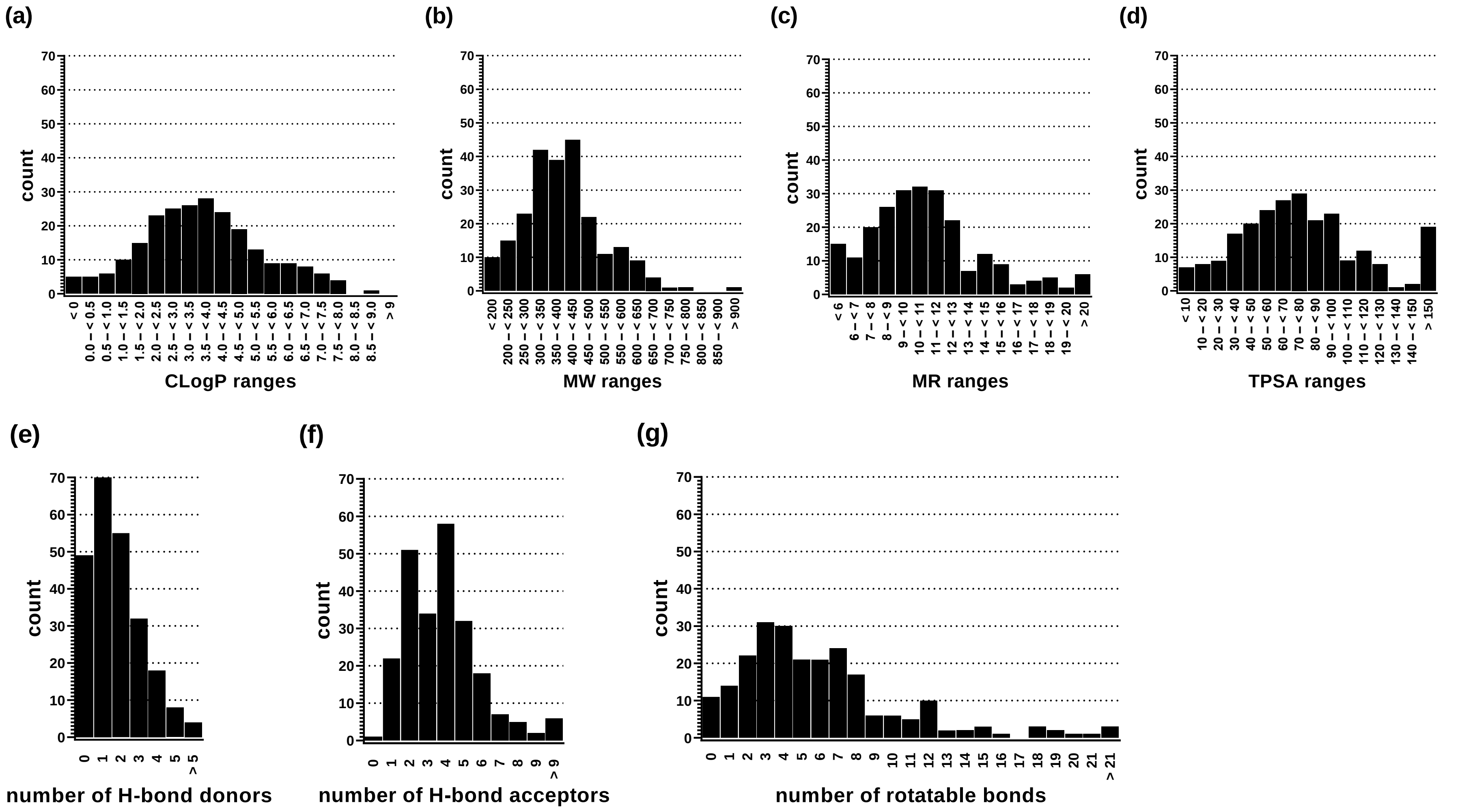


**Fig. S17** Distribution of physicochemical and molecular-structural attributes of the 236 early drug development stage molecules of the HD_BPMDS as determined by MOE version 2019.01. **a** Calculated octanol-water partition coefficient (CLogP). **b** Molecular weight (MW)**. c** Molar refractivity (MR). **d** Topological polar surface area (TPSA). **e** H-bond donors. **f** H-bond acceptors. **g** Rotatable bonds.

***Advanced drug development stage molecules (in vivo, etc.):***

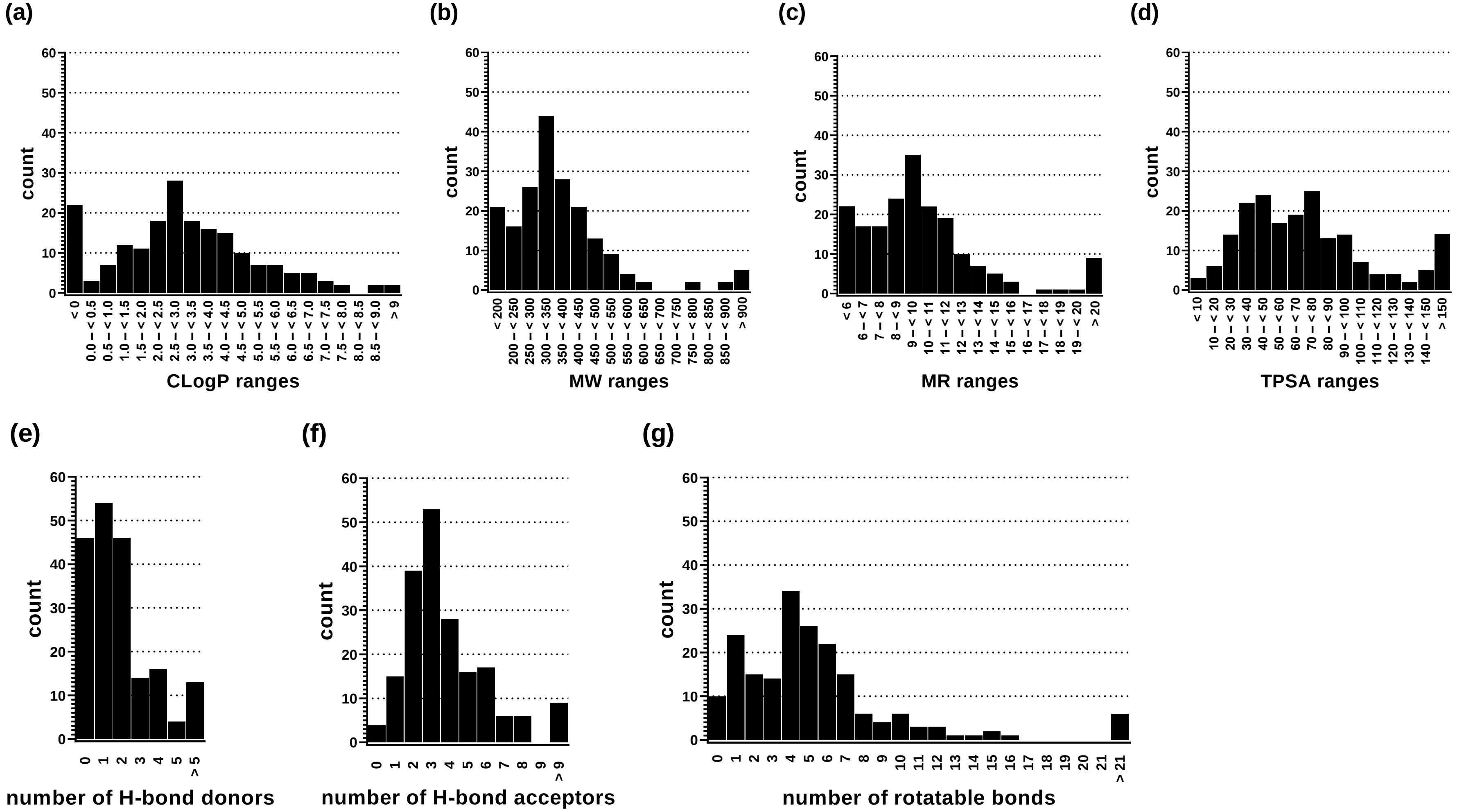


**Fig. S18** Distribution of physicochemical and molecular-structural attributes of the 193 advanced drug development stage molecules of the HD_BPMDS as determined by MOE version 2019.01. **a** Calculated octanol-water partition coefficient (CLogP). **b** Molecular weight (MW)**. c** Molar refractivity (MR). **d** Topological polar surface area (TPSA). **e** H-bond donors. **f** H-bond acceptors. **g** Rotatable bonds.
